# Senescence-associated alterations in histone H3 modifications, HP1 alpha levels and distribution, and in the transcriptome of vascular smooth muscle cells in different types of senescence

**DOI:** 10.1186/s12964-025-02315-8

**Published:** 2025-07-01

**Authors:** Agnieszka Gadecka, Marta Koblowska, Helena Kossowska, Roksana Iwanicka-Nowicka, Dorota Janiszewska, Grażyna Mosieniak, Krzysztof Bojakowski, Krzysztof Goryca, Anna Bielak-Zmijewska

**Affiliations:** 1 Laboratory of Molecular Basis of Aging, Nencki Institute of Experimental Biology, Polish Academy of Sciences, 3 Pasteur St., Warsaw, 02-093 Poland; 2 Laboratory of Systems Biology, Faculty of Biology, University of Warsaw, 1 Miecznikowa St., Warsaw, 02-096 Poland; 3 Institute of Biochemistry and Biophysics, Polish Academy of Sciences, 5A Pawińskiego St., Warsaw, 02-106 Poland; 4 Laboratory of Calcium Binding Proteins, Nencki Institute of Experimental Biology, Polish Academy of Sciences, 3 Pasteur St., Warsaw, 02-093 Poland; 5 Laboratory of Cytometry, Nencki Institute of Experimental Biology of Polish Academy of Sciences, 3 Pasteur St., Warsaw, 02-093 Poland; 6 Department of General and Vascular Surgery, National Medical Institute of the Ministry of the Interior and Administration, 137 Wołoska St., Warsaw, 02-507 Poland; 7 2nd Department of Vascular Surgery and Angiology, Centre of Postgraduate Medical Education, 137 Wołoska St., Warsaw, 02-507 Poland; 8 Genomics Core Facility, Centre of New Technologies, University of Warsaw, 2c Banacha St., Warsaw, 02-097 Poland

**Keywords:** Senescence, VSMCs, Chromatin structure, Histone H3 modifications, HP1α

## Abstract

**Background:**

Cellular senescence is a fundamental process leading to organismal aging and age-related diseases. Alterations accompanying cellular senescence concern, among others, nucleus architecture, chromatin structure, DNA damage and gene expression. Some changes are universal for all types of senescence, but some characteristics are typical for a given senescence inductor or cell type. The aim of the study was to analyze senescence-associated alterations in chromatin modifications and look for differences depending on senescence type (replicative, RS and stress-induced premature senescence, SIPS) in vascular smooth muscle cells (VSMCs) in vitro. The alterations were compared with those observed in VSMCs derived from atherosclerotic plaques (ex vivo) and, to assess their universality, with those in senescent fibroblasts.

**Methods:**

We investigated the level and distribution of HP1α and H3 modifications that are markers of hetero- and euchromatin (H3K9me3, H3K27me3, H3K4me3, H3K9Ac - WB and IF), alterations in the transcriptomic profile (DNA microarray, qPCR), H3K4me3, H3K9me3 and HP1α protein distribution in the genome (ChIP-seq), and expression of enzymes involved in histone post-translational modifications (DNA microarray, qPCR, WB, IF).

**Results:**

Our results have shown that the decline in H3K4me3 and H3K9me3 modifications and in HP1α is a universal hallmark of senescence in all tested cell and senescence types, although the extent of the change depends on the senescence inductor. The distribution of H3K4me3 and H3K9me3 in the genome of VSMCs depends on the senescence type, and the transcriptomic analysis identified genes and processes specific to each type.

**Conclusions:**

We characterized senescence and cell type-dependent changes in chromatin-associated proteins and enzymes involved in histone H3 decoration which, in consequence, impact senescence-associated gene expression. We can conclude that certain similar alterations occur in senescent VSMCs ex vivo, although inter-individual differences usually obscure them. Our results clearly showed that differences existed not only between young and senescent cells but also between SIPS and RS ones. The subtle differences between various SIPS types suggest that various stressors activate the same cellular mechanisms. This study can serve as a starting point to search for factors that may be used to distinguish between SIPS and RS, which in turn could be helpful in defining conditions responsible for accelerated aging.

**Supplementary Information:**

The online version contains supplementary material available at 10.1186/s12964-025-02315-8.

## Introduction

In the face of an increased population of people 65 + and, consequently, an increased number of people suffering from aging-related diseases, the search for ways to delay the aging process has become an essential branch of biogerontology. A breakthrough was the demonstration that senescent cells are directly responsible for the aging of the organism and the development of age-related diseases, including cardiovascular diseases such as atherosclerosis and hypertension [1–3]. Elimination of senescent cells from tissues and organs has proven to be a promising rejuvenating mechanism [1–2]. However, no adequate and safe method of carrying out such a procedure in humans has been developed till now. Research concerning the recognition of senescence mechanisms, alterations and specific markers is constantly under way. Such research may contribute to our understanding of the aging process, identification of new anti-senescence targets and implementation of strategies to alleviate its effects.

Cellular senescence is related to permanent cell cycle arrest while preserving metabolic functions. It can occur by exhaustion of the cell’s ability to divide (replicative senescence - RS) or as a result of stress (stress-induced premature senescence – SIPS/PS) [4]. Senescence entails a number of morphological changes, alterations in nuclear architecture, chromatin structure and gene expression. A gradual decrease in condensed, transcriptionally inactive heterochromatin content in favor of transcriptionally active euchromatin is observed. This could be caused by the reduced synthesis of histones, disturbance of the balance between repressive and activating post-translational histone modifications and impaired activity of enzymes involved in histone PTMs (Post-translational modifications) or proteins stabilizing the chromatin structure. One such protein is HP1α (Heterochromatin Protein 1 subunit α), which, by attaching to trimethylated lysine 9 of histone 3 (H3K9me3), prompts chromatin condensation, stabilization and gene silencing [5]. The role of HP1α in senescence is still not fully elucidated. We aimed to study the alterations in HP1α protein level, selected histone H3 modifications and enzymes responsible for histone H3 decoration in vascular smooth muscle cells (VSMCs) subjected to various types of senescence (in vitro study) and their impact on chromatin structure and gene expression. We used three different models of senescence, namely RS and PS, the latter induced by two different mechanisms: DNA damage-dependent and DNA damage-independent [6–8]. Since VSMCs are strongly involved in atherosclerosis development and progression, the results were verified in VSMCs derived from atherosclerotic plaque (AP, ex vivo study). The universality of the alterations was analyzed using senescent fibroblasts (in vitro study). Our comprehensive analysis of the nucleus and chromatin structure in senescent VSMCs revealed several significant changes differentiating RS from PS. We have observed a significant decrease in H3K9me3, H3K4me3 and HP1α levels and reorganization of HP1α in the nucleus (formation of many distinct foci) associated with senescence. No significant senescence-associated alterations were detected in H3K9Ac and H3K27me3. Furthermore, it has been demonstrated that the decline in H3K4me3, H3K9me3 and HP1α is a universal hallmark of senescence in all tested cell and senescence types, although the extent of reduction is different in PS and RS. As regards AP cells, the level of tested modifications was heavily dependent on the donor and the percentage of senescence cells in the whole cell population. ChIP-seq (chromatin immunoprecipitation followed by sequencing) data showed that in VSMCs, the distributions of H3K4me3 and H3K9me3 in the genome were senescence type-dependent. Transcriptomic analysis identified genes specific to each type of senescence and pointed out processes that were altered in particular types. This study provides an extensive characterization of senescence-related changes and demonstrates that alterations in chromatin structure depend on the type of senescence and correlate with a decrease in histone H3 methylation of lysines 9 and 4, which can be related to changes in enzymes introducing these modifications. HP1α reduction is a universal feature of senescence and does not seem to be helpful in distinguishing between senescence types. However, based on the results, new markers of senescence, anillin (ANLN) and claudin-1 (CLDN1) could be proposed [submitted], of which the first one may serve as a marker of cells that ceased proliferation, and the second one might be useful for distinguishing RS from PS in VSMCs.

## Materials and methods

### Cell cultures

Human vascular smooth muscle cells (VSMCs), derived from aorta of at least 3 young male donors (age 29–34 years), were purchased from ATCC or Gibco and cultured in Vascular Cell Basal Medium (ATCC) supplemented as defined by the manufacturer in a humidified atmosphere with 5% CO_2_ at 37^o^C. The cells were seeded at 4,000 cells/cm^2^ density and passaged when they reached 70–80% confluency until they reached the state of replicative senescence (cells were recognized as senescent if at least 70% of the population were SA-β-gal-positive and no more than 30% were BrdU-positive). Premature senescence was induced in cells on passage 10 or lower by treatment with curcumin (7,5 µM concentration, DNA-damage independent senescence) or doxorubicin (100 nM concentration, DNA-damage dependent senescence) as previously described by us [6–8]. Briefly, to induce senescence, VSMCs were treated 24 h after seeding and analyzed after 7 days of culture with the drug. Replicatively senescent cells were analyzed 24 h after seeding. Cells at early passages were collected 24 h after seeding and served as a control. The efficiency of senescence induction is shown in the Additional files (Additional file 1).

VSMCs derived from atherosclerotic plaques (AP) were obtained from patients who underwent carotid endarterectomies. The explants (1–2 mm^2^ fragments) were placed in DMEM on selective dishes covered with gelatin (Biocoat Gelatin Cellware, Corning) and they remained in culture until cells reached confluency, no more than 30 days. Next, cells were detached from the culture dish, seeded at a density of 4,000 cells/cm^2^ in Vascular Cell Basal Medium, cultured for 24 h, and collected for subsequent analysis. The proliferation potential of cells derived from atherosclerotic plaques is presented in Additional file 1. Cells from atherosclerotic plaques were compared to established VSMC senescence models.

Human skin fibroblasts were purchased from ATCC or Gibco and cultured in DMEM (Sigma-Aldrich or Merck) supplemented with 10% fetal bovine serum (FBS, BioWest), antibiotic and antimycotic mix solution (100 U/ml penicillin, 100 µg/ml streptomycin and 250 ng/ml amfoterycin B, Sigma-Aldrich). Cells were seeded at 4,000 cells/cm^2^ density and passaged when they reached 70–80% confluency until replicative senescence (evaluated in similar manner as in VSMCs). Premature senescence induction was performed in cells on passage 25 or lower in the same manner as in VSMCs; however, different concentrations of curcumin (10 µM) or doxorubicin (150 nM) were used due to lower sensitivity of these cells, which was established experimentally (Additional file 2). Fibroblasts were treated 24 h after seeding and analyzed after 7 days of culture with the drug. Replicatively senescent cells were analyzed 24–48 h after seeding. Young cells collected 24–48 h after seeding, depending on the assay, served as control (detailed description in subchapter 3.8 and Additional file 2).

### Immunocytochemistry

Cells were fixed with 4% PFA and stored in ethanol at -20^o^C. Cells were permeabilized with 0.5% Triton X-100 for 10 min, blocked in 1.5% Goat Serum, 2% Bovine Serum Albumin and 0.5% Triton X-100 for 10 min and incubated with primary antibody for 2 h. Primary antibodies used: H3K9me3 (Diagenode, C15410193, 1:500), H3K27me3 (Diagenode, C15410195, 1:500), H3K4me3 (Diagenode, C15410003, 1:200), H3K9Ac (Cell Signaling, 9649, 1:400). Subsequently, cells were incubated with secondary antibody Alexa Fluor 488 or Alexa Fluor 555 (ThermoFisher Scientific, 1:500) for 1 h. DNA was stained with DAPI (1 µg/ml). Cells were analyzed using a fluorescent microscope Nikon Eclipse Ti.

### Western blotting

Whole-cell protein lysates were prepared according to Laemmli [9]. Electrophoretically separated proteins were transferred to a nitrocellulose membrane and blocked in 5% BSA or low-fat milk. Incubation with primary antibodies was conducted overnight at 4^o^C. Primary antibodies used: HMGB1 (Abcam, ab79823, 1:500), lamin B1 (Santa Cruz, sc-365962, 1:500), HP1α (Cell Signaling, #2616, 1:1000), H3K4me3 (Diagenode, C15410003, 1:1000), H3K9me3 (Diagenode, C15410193, 1:1000), H3K9Ac (Cell Signaling, #9649, 1:1000), H3K27me3 (Diagenode, C15410195, 1:1000), GAPDH (Millipore, MAB374, 1:150000), Suv39H1 (Cell Signaling, 8729, 1:500), EED (GeneTex, GTX634650, 1:1000), H3 (Abcam, ab1791, 1:10000), H3.3 (Abcam, ab176840, 1:1000). Membranes were then incubated with HPR-conjugated secondary antibody at 1:2000 dilution in low-fat milk (Dako, Agilent), washed and developed with ECL (ThermoFisher Scientific). Protein level was normalized to GAPDH or H3 and presented as a fold change relative to control.

### RNA isolation

Trypsinized VSMCs and AP cells were fixed in RNAlater solution and stored in -80 °C. Isolation of total RNA was performed in an automated manner using the MagNA Pure Total RNA Isolation kit (Roche) in the MagNA Pure Compact apparatus (Roche) according to the manufacturer’s instructions. The RNA concentration and quality were measured using a NanoDrop 2000 spectrophotometer (ThermoFisher Scientific), while integrity was determined using capillary electrophoresis with a Bioanalyzer 2100 (Agilent Technologies) and a set of dedicated reagents provided by RNA 6000 Nano kit (Agilent Technologies). Isolated RNA was stored at -20 °C (short-term) or -80 °C (long-term). To control the level of contamination with genomic DNA, a PCR reaction was performed to amplify exons 1 and 2 of human β-globin (HBB). The reaction involved PCR reaction buffer (A&A Biotechnology), an appropriate pair of HBB primers (sequence shown in Additional file 5) and the tested sample. The reaction was performed in a Mastercycler^®^ nexus (Eppendorf) under the following conditions: 4 min at 94^o^C, 30 cycles of 45 s at 94^o^C, 45 s at 57^o^C, 1 min 72^o^C. The obtained product was examined using electrophoresis in a 1% agarose gel.

### Transcriptomic profile analysis using DNA microarrays

The gene expression profile of VSMCs subjected to different types of senescence (in at least three biological replicates) was examined by microarray analysis using 250 ng of RNA and the GeneChip™ WT PLUS reagent kit (ThermoFisher Scientific) compatible with the Affymetrix Gene Atlas System platform, according to the manufacturer’s instructions. The prepared samples were hybridized to Affymetrix™ HuGene 2.1 ST Array Strips (Affymetrix, Santa Clara, CA, USA). After washing and staining, the arrays were scanned in the GeneAtlas Imaging Station (Affymetrix), generating. CEL files as the data output. Data was analyzed using Transcriptome Analysis Console (TAC) software (ThermoFisher Scientific). Probes signal intensities were normalized with RMA (Robust Mulit-array Average) method and, after quality control, one-way ANOVA was applied to determine differentially expressed genes (DEGs) between treated cells and control. To minimize the variability originating from different sample preparation dates in a comparison analysis, batch effect in TAC was applied. The criteria for selecting DEGs were fold change ≤ − 1.3 and ≥ 1.3, and p value ≤ 0.05. The enrichment analysis was performed using g: Profiler tool [10, 11] with default parameters.

### Analysis of mRNA levels using real-time PCR (qPCR)

The reverse transcription reaction was performed using the Maxima First Strand cDNA Synthesis Kit (ThermoFisher Scientific) according to the manufacturer’s instructions. The reaction was performed in a Mastercycler^®^ nexus thermal cycler (Eppendorf) using 1 µg of RNA isolated as described in 2.4. Reaction conditions consisted of 10 min at 25 °C, 15 min at 50 °C and 5 min at 85 °C. The expression of selected genes changing during senescence was assessed by performing real-time polymerase chain reaction (qPCR). For this purpose, the LightCycler^®^ 480 SYBR Green I Master reagent (Roche), a set of previously validated primers purchased from realtimeprimers.com (primer sequences are shown in Additional file 5), and 20 ng of cDNA template (VSMCs and AP cells) were used. The reaction was performed in triplicates in a volume of 10 µl using the LightCycler^®^480 apparatus/instrument (Roche) under the following conditions: 10 min at 95 °C and 40 cycles of 10 s at 95 °C, 10 s at 59 °C and 30 s at 72 °C. GAPDH, EIF2B and DAD1 were used as reference genes and changes in gene expression levels were analyzed using the Pfaffl method [12] and were presented as a fold change relative to control cells.

### Analysis of the enrichment of H3K4me3, H3K9me3 and HP1 alpha protein in the genome by chromatin Immunoprecipitation and next-generation sequencing (ChIP-seq)

The chromatin immunoprecipitation procedure was performed using the iDeal kit for histones (Diagenode) according to the manufacturer’s instructions. Briefly, 3 million VSMCs were trypsinized, suspended in PBS, and subjected to the cross-linking process with fresh, 1% methanol-free formaldehyde solution (Carl Roth) at room temperature. The time of reaction depended on the experimental variant (after the optimization process). Control cells were incubated for 1 min, cells treated with doxorubicin and curcumin for 2 min, and replicatively senescent cells for 3 min. The reaction was stopped by adding 0.125 M glycine and further incubation at room temperature for 5 minutes. Cells were then washed twice with cold PBS and centrifuged at 1800 rpm (Eppendorf 5424 R) for 5 min at 4 °C. From this point on, the entire immunoprecipitation procedure was performed at 4 °C using cold reagents. After cell lysis, chromatin was sheared using Bioruptor Plus (Diagenode) with a built-in cooling system set at 4 °C. Cells were sonicated at high power mode for 40 cycles (30s ON, 30s OFF) (CTRL) or 30 cycles (DOX, CUR, and RS). Sonication efficiency was controlled using 2100 Bioanalyzer and the High Sensitivity DNA kit (Agilent). The chromatin immunoprecipitation was performed overnight on a rotating wheel using 7.5 × 10^5^ cells, dedicated buffers, A-coated beads and antibodies recognizing H3K4me3 (1 µg, Diagenode, C15410003), H3K9me3 (1.5 µg, Diagenode, C15410193) and HP1α (2.5 µg, Cell Signaling, #2616). After several subsequent washings, chromatin was eluted and decrosslinked overnight at 65 °C with shaking. Finally, DNA was precipitated with magnetic beads and quantified using Qubit dsDNA HS kit (Invitrogen). Immunoprecipitation was assessed by qPCR using LightCycler^®^ 480 SYBR Green I Master (Roche) and the following amplification conditions: 10 min at 95 °C and 40 cycles of 30 s at 95 °C, 30 s at 60 °C, 30 s at 72 °C.

The library was prepared from 3 ng of material using the KAPA HyperPrep Kit and compatible KAPA Unique Dual-Indexed Adapter Kit (KAPA Biosystems) according to the manufacturer’s instructions. The library quality was assessed using 2100 Bioanalyzer and a High Sensitivity DNA Analysis kit (Agilent), and the quantity was measured using qPCR using the KAPA Library Quantification kit (KAPA Biosciences).

Raw reads were trimmed using Trimmomatic software [13]. Trimmed sequences were mapped to the human reference genome provided by ENSEMBL, (GRCh38) using Bowtie2 [14] with default parameters. Duplicates reads were identified and removed using the MarkDuplicates tool from the GATK package (version 4.2.3.0) [15] with default parameters except OPTICAL_DUPLICATE_PIXEL_DISTANCE set to 12,000. Peaks were detected with MACS2 [16]. To account for variability between replicates, reads overlapping each peak detected by MACS2 were counted, separately in each replicate, with the summarize Overlaps tool from the Genomic Alignments library. Resulting counts were used by edgeR [17] to select locations with reproducible binding patterns across all replicates. Peaks were visualized using the IGV software. Functional analysis was performed using g: Profiler tool with default settings.

### Analysis of enzyme activity of histone deacetylases

The enzymatic activity of class I, IIb and IV histone deacetylases was determined colorimetrically using the Colorimetric Histone Deacetylase Activity Assay kit (ScienCell). During the first stage of reaction, lysine residues in the substrate included in the kit are deacetylated by HDAC enzymes. The resulting product releases a chromophore detected at 405 nm after incubation in a dedicated development buffer. Histone deacetylase activity was given in pmol of deacetylated product/min/mg of lysate using formulas provided by the manufacturer. The set of tested deacetylases included HDAC1, HDAC2, HDAC3, HDAC6, HDAC8, HDAC10 and HDAC11. Whole-cell protein extracts were obtained using RIPA lysis buffer (Sigma) supplemented with protease inhibitors (Roche). Protein concentration was estimated using the BCA colorimetric method (Sigma); absorbance was measured at 562 nm (Tecan). The results were compared to a standard curve.

### Senescence-associated β-Galactosidase activity

Enzyme activity was analyzed according to Dimri [18]. Cells were fixed (2% formaldehyde, 0.2% glutaraldehyde, PBS), washed and incubated overnight at 37^o^C without access to light and carbon dioxide in staining buffer (5 mM potassium ferrocyanide, 5 mM potassium ferrycyanide, 150 mM NaCl, 2 mM MgCl2, 0.02 M phosphate buffer and X-Gal at concentration 1 mg/ml, pH 6.0.). Nuclei were stained with DAPI (1 µg/ml). Cells were analyzed using a fluorescent microscope Nikon Eclipse Ti. The results are expressed as % of cells with increased enzyme activity (blue color) to all cells (based on DAPI-stained nuclei).

### Bromodeoxyuridine (BrdU) incorporation assay

The ability of cells to replicate DNA was assessed using the BrdU incorporation assay. BrdU (Sigma-Aldrich) at 10 µM concentration was added for 24 h to the medium. Then, cells were fixed with ice-cold 70% ethanol and stored at -20^o^C for a minimum of 24 h. Cells were immunostained with anti-BrdU primary antibody (1:100, Becton Dickinson) and a secondary Alexa Fluor 488-conjugated antibody (1:500, ThermoFisher Scientific). DNA was stained with DAPI (1 µg/ml). The percentage of dividing cells was assessed by considering the number of BrdU-positive cells among all cells (DAPI-stained nuclei).

### Cytometric analysis of the cell cycle

For DNA content analysis, VSMCs and fibroblasts were fixed in 70% cold (-20^o^C) ethanol and stored at least 24 h at -20ºC. After washing twice in a 0,05% Tween solution in PBS (1200 rpm, 5 min., Eppendorf 5702) the pellet was resuspended in PBS 1:1 with citrate buffer (4 mM citric acid and 0,2 M Na2HPO4) and incubated for 5 min. After centrifugation (1200 rpm, 5 min), cells were resuspended in staining buffer (3,8 mM sodium citrate, 50 µg/ml RNAse A, 50 µg/ml PI, in PBS) and incubated for 30 min in the dark. Approximately 10,000 cells were analyzed using the LSRFortessa flow cytometer (BD Biosciences).

### Statistical analysis

Statistical analysis was performed on at least three separate biological replicates. Standard deviations were determined using the GraphPad Prism program, and in most cases, one-way ANOVA was used to determine the statistical significance of changes occurring between senescent and young cells. If another statistical test is used, its details are included in the description of the given method and in the figure caption. The obtained values were recorded as follows: *p* < 0.05 (*), *p* < 0.01 (**), *p* < 0.001 (***), *p* < 0.0001 (****). The following software were used to prepare the figures: ImageJ, GraphPad, Excel, Transcriptome Analysis Console.

## Results

### The distribution and amount of histone H3 modifications are altered in all types of senescence but to a different extent

**Fig. 1:**
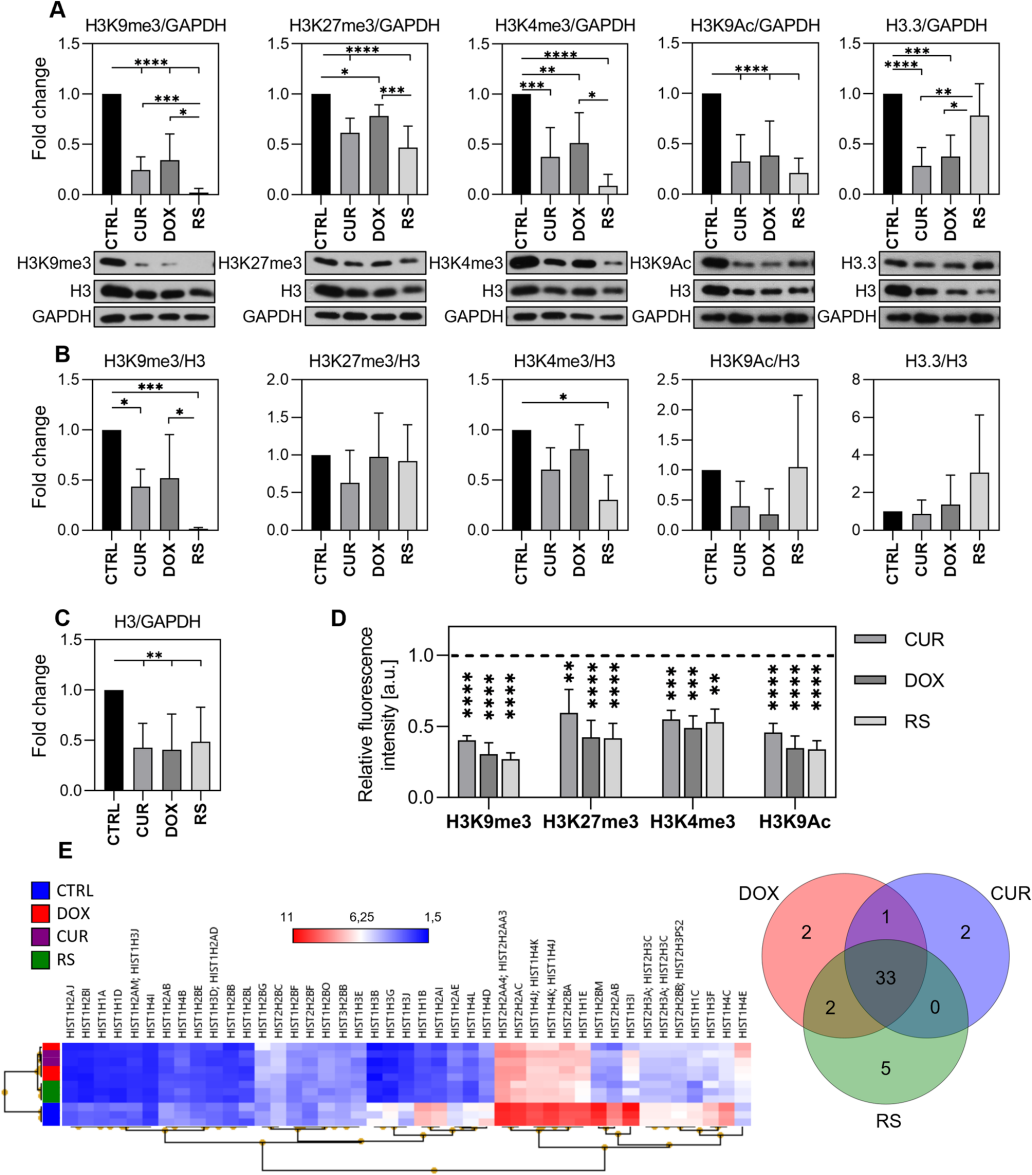
Alterations in the level of histone H3 modifications and nucleosome composition in premature (CUR, DOX) and replicative senescence (RS). (**A**) The protein levels of selected heterochromatin (H3K9me3, H3K27me3) and euchromatin (H3K4me3, H3K9Ac) markers and H3.3 were analyzed by Western blotting and protein expression data was normalized to GAPDH or (**B**) to H3. (**C**) total histone H3 were analyzed by Western blotting and normalized to GAPDH. (**D**) Immunofluorescence staining expressed as relative fluorescence intensity. (**E**) Changes in transcript level of various histone variants was analyzed with microarrays where the Venn diagram on the left shows the number of histone genes per each group/treatment relative to control cells and the heatmap shows hierarchical clustering of genes encoding clusters of histone variants as signal changes (log2). The mean fluorescence signal was recorded for cells based on their nuclear surface area (for young cells < 200 µm^2^, for senescent cells > 200 µm^2^) (**D**). Densitometric data and mean fluorescence intensity signal detection were calculated as a mean ± SD from three independent experiments in cells isolated from at least three donors (*n* ≥ 3) (**A**, **B**, **C**). Results are presented as a relative fold change compared to control cells. The dashed line represents control cells (**D**). Venn diagram and heatmap were prepared using TAC software. Statistical analysis was performed using one-way ANOVA between all experimental variants: *p* < 0,05 (*), *p* < 0,01 (**), *p* < 0,001 (***), *p* < 0,0001 (****)

In VSMCs undergoing senescence in three different manners (premature induced by curcumin - CUR or doxorubicin - DOX and replicative - RS), we analyzed histone H3 methylation and acetylation, key epigenetic modifications that significantly influence chromatin packing and gene expression. Modifications typical of condensed chromatin - heterochromatin, i.e. H3K9me3 and H3K27me3, and markers typical of active euchromatin, such as H3K4me3 and H3K9Ac were selected (Fig. 1). To ensure that changes in the amount of a given modification were not due to alterations in H3 level, we normalized the protein level to both GAPDH (unchanged in all variants) and H3 (the expression of which diminished during senescence, Fig. 1C). Considering heterochromatin markers, we observed more than a 50% decrease in H3K9me3 in PS (CUR, DOX) and its almost complete disappearance in RS (Fig. 1A). This decrease was evident when compared to both GAPDH and H3 level (Fig. 1B). A small decrease was recorded for H3K27me3 when normalized to the GAPDH level, but not if normalized to the H3 level and was similar in both types of senescence. Although chromatin loosening was linked to a reduction in modifications characteristic of heterochromatin, it did not coincide with an increase in modifications typically associated with active euchromatin. Unexpectedly, the levels of H3K4me3 also decreased in both types of senescence (normalization to both GAPDH and H3). H3K4me3 showed an identical profile to H3K9me3, and significant differences between PS and RS were also visible. The H3K9Ac level did not change if normalized to H3 and diminished when GAPDH served as loading control. The alterations were senescence-type independent. We analyzed the chromatin compaction markers by immunocytochemical staining (Fig. 1D). The fluorescence intensity was measured only for nuclei with a surface area greater than 200 µm^2^ for senescent cells or smaller than 200 µm^2^ in the case of young ones. Images of DAPI-stained nuclei were analyzed using CellProfiler, and the nuclear surface area was among the measured parameters. After statistical analysis, the data were visualized using box plots to compare distributions across experimental conditions.

To define a threshold for further analysis, we used the lower edge of the box (Q1) in the CUR condition, which was 200 μm². This value reflects the lower boundary of typical nuclear sizes where curcumin-induced changes were most evident. Notably, it approximately aligns with the upper whisker (highest non-outlier value) of the control condition, supporting its use as a biologically relevant cut-off to identify senescent cells with enlarged nuclei (manuscript with details is in preparation). The results showed that the average staining intensity for all studied modifications decreased.

In addition to a significant loss of histone modifications, H3K9me3, H3K4me3 and histone H3 itself, in both types of senescence, the H3.3 variant (normalized to GAPDH) decreased in PS cells, while RS cells exhibited H3.3 levels comparable to young cells. However, if H3 served as control, no changes were observed between young and PS cells and even an increase in RS cells was noticed (without statistical significance) (Fig. 1A and B). The level of histone H3.3, which is incorporated in a DNA replication-independent manner during transcription, was analyzed due to the association of senescence with cell cycle arrest [17, 19]. H3.3 plays a role in chromatin organization and function and regulates gene expression and genome integrity. It is involved in both transcriptional activation and repression, depending on the genomic location and specific histone chaperones [20, 21]. Microarray data revealed that senescence is associated with the transcript loss of numerous canonical and non-canonical histone variants, regardless of the type of senescence. (Fig. 1E). The observed decrease in expression of genes encoding histone clusters may lie behind senescence-associated chromatin loosening. Our results indicated that H3K9me3 and H3K4me3 decreased in all types of senescence while H3K27me3 and H3K9Ac were not altered.

### Distribution of H3K4me3 and H3K9me3 in the genome differ in young and senescent VSMCs and depend on the senescence inductor

Due to the gradual decline in H3K4me3 and H3K9me3 in the examined types of senescence, we analyzed where these markers accumulate in the genome and how this may affect the gene expression profile. We analyzed the sites of their deposition using histone immunoprecipitation and next-generation sequencing (ChIP-seq).

**Fig. 2:**
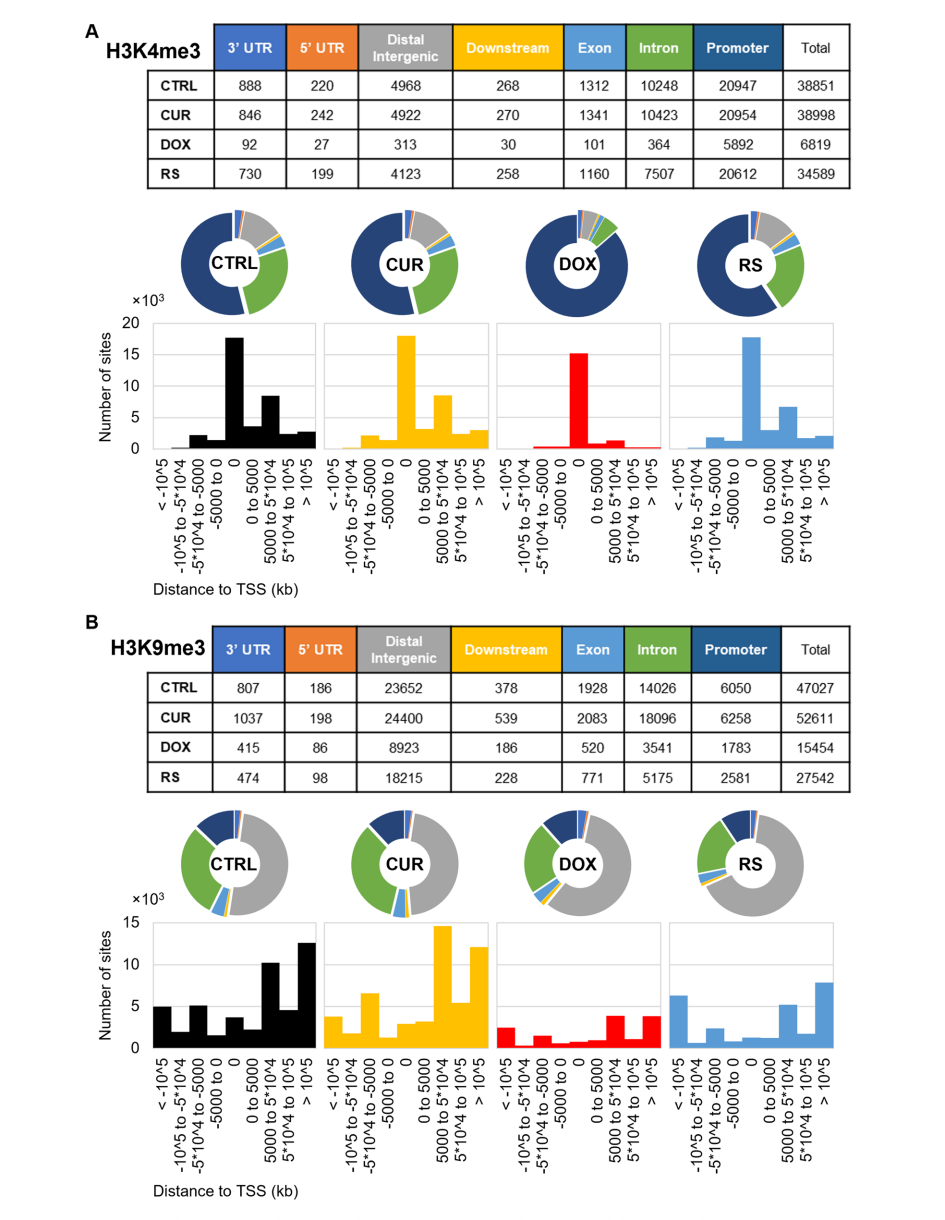
Distribution of H3K4me3 (**A**) and H3K9me3 (**B**) in the genome of young and senescent VSMCs. Tables summarize the enrichment of H3K4me3 and H3K9me3 in given areas of the genome for each experimental variant. Pie charts show the percentage amount of a given modification in each area, and the bar charts below show the distribution of enrichment sites around the transcription start site (TSS). The following labels were used: black - CTRL, yellow - CUR, red - DOX, blue – RS and colors in the table correspond to colors applied for pie charts

Cells treated with doxorubicin (DOX) had the lowest number of H3K4me3 reads, and the total number of enrichment regions was 5-fold lower than in other variants (Fig. 2A). The number of reads, above 30,000, was similar in CTRL, CUR, and RS. Most of the enrichment was recorded at gene promoter regions at the transcription start site (TSS), which is consistent with the literature data for this modification [22]. Exceptionally, in the DOX variant, over 80% of all reads were located mainly in the promoters, and the small spread around the TSS may suggest the appearance of a very narrow peak of enrichment for this histone modification.

For H3K9me3, the lowest number of reads was also recorded for the DOX variant (15454). Slightly more were obtained for RS (27542) and CTRL (47027) cells, while, the highest number of enrichments/reads was obtained for cells treated with curcumin (52611) (Fig. 2B). Moreover, H3K9me3 localized mainly to distal intergenic regions for all variants tested. Occasionally, this modification accumulated at promoter sites, but this was only 10% of all reads, and TSSs were mostly devoid of H3K9me3. In addition, the visual enrichment analysis in IGV software showed a slightly higher density of this modification in pericentromeric and subtelomeric regions (Additional file 3).

### Functional analysis of the enrichment sites in H3K4me3 and H3K9me3 showed senescence-type dependent differences

**Fig. 3:**
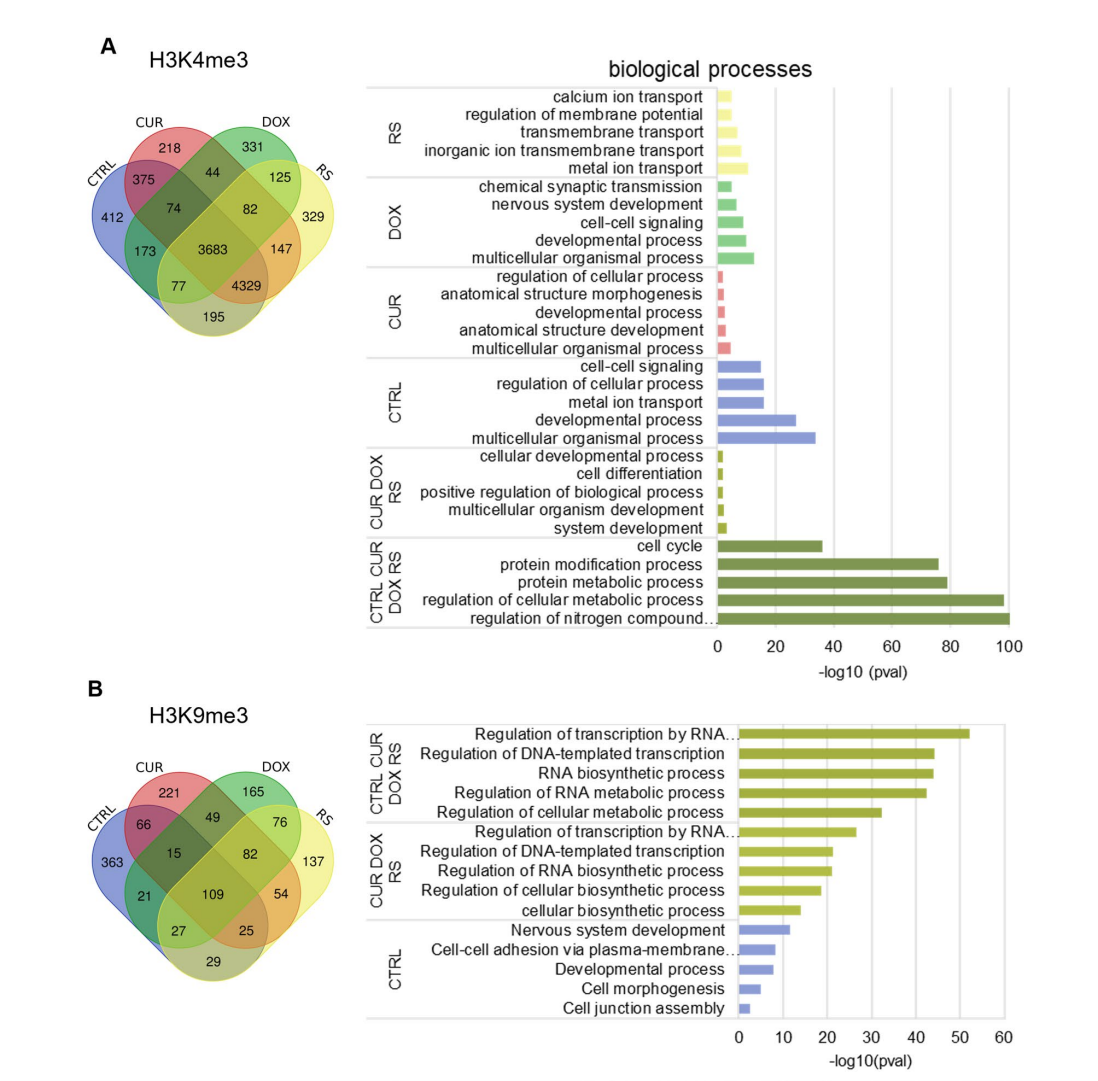
Functional analysis of H3K4me3 (**A**) and H3K9me3 (**B**) enrichment in biological processes. The Venn diagram shows intersections of differentially expressed genes for all experimental variants, and the bar charts show biological processes in which the transcriptionally active (enrichment in H3K4me3) or silenced (enrichment in H3K9me3) genes are involved. Gene sets were analyzed using g: Profiler and Gene Ontology database

Significant reads indicated by edgeR (edgeR < 0.05), located in the promoters of protein-coding genes in all experimental variants, were selected for functional analysis of H3K4me3-rich regions (Fig. 3A). As H3K4me3 usually marks active chromatin, its enrichment in the gene promoter is strongly associated with gene transcription and as a result, activation of biological processes the genes are involved in. The Venn diagram shows intersecting genes with H3K4me3 located in their promoters in both young and senescent cells (i.e. CTRL, CUR, DOX, RS) that were used to identify enriched functional terms from Gene Ontology. The set of genes common to senescence, i.e. the intersection of the CUR, DOX and RS sets, contained 82 genes responsible for development and cell differentiation. However, the presence of only 44 genes characteristic of PS in both doxorubicin (DOX) and curcumin (CUR) treated cells hindered a comprehensive functional analysis. Conversely, the appearance of enriched H3K4me3 peaks at *loci* specific to a given experimental condition (CTRL: 412, CUR: 218, DOX: 331, and RS: 329) indicates genome-wide reorganization of H3K4me3. The results of functional enrichment analyses for key processes associated with these genes, along with other overlapping gene sets are shown in the graph (Fig. 3A).

Functional enrichment analysis of GO terms connected with protein-coding genes decorated with inactive chromatin mark, H3K9me3, in promoter regions, introns, or intergenic sites was also performed using significant reads (edgeR < 0.05). Due to an insufficient number of reads, a complete analysis of the same groups as in H3K4me3 was not performed (Fig. 3B). Moreover, despite an extensive set of characteristic enrichment sites for CUR (221), DOX (165) and RS (137), no information was obtained on the biological processes in which the genes might be involved.

### The expression and activity of enzymes regulating histone modifications varies according to senescence type

The observed decline in H3K4me3 and H3K9me3 in senescent cells has prompted us to investigate the involvement of selected histone-modifying enzymes (methyltransferases, demethylases, but also acetyltransferases and deacetylases) specific for lysines 4, 9 and 27 of histone H3. The corresponding list of proteins was downloaded from Gene Ontology database and compared with gene expression data obtained with DNA microarrays. The summary of the gene expression profile for each analyzed enzyme and the applied GO term ID are presented in Additional file 6.

#### Histone H3 methyltransferases (HMTs)

##### H3K9 HMTs:

**Fig. 4:**
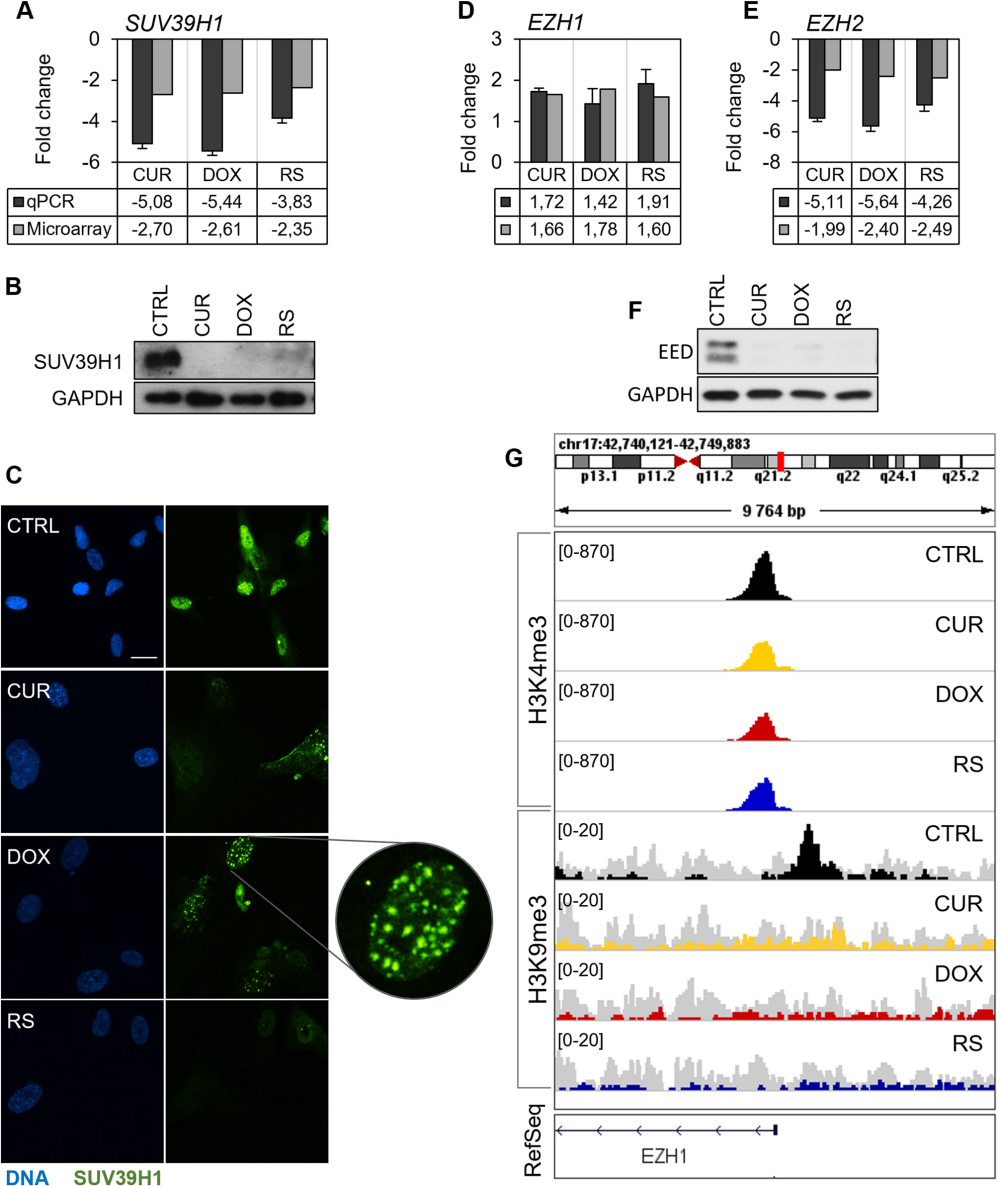
Analysis of selected methyltransferases in different types of senescence in VSMCs. (**A**) Changes in SUV39H1 mRNA level were measured with qPCR (black) and microarrays (gray). Data are presented as a mean ± SD fold change relative to control (*n* = 3). (**B**) Protein level of SUV39H1 analyzed by Western blot. Representative image from 3 independent experiments. (**C**) Reorganization of SUV39H1 protein in the nucleus visualized by immunocytochemical staining. 10 μm scale; (**D**, **E**) Gene expression profile of EZH1 and EZH2, respectively. (**F**) Protein EED expression was evaluated by Western blotting. Representative images from 3 independent experiments. (**G**) Visual representation of enrichment profile of H3K4me3 and H3K9me3 in the gene EZH1 promoter region generated in IGV. Color scheme: black – control, yellow – curcumin-treated cells, red – doxorubicin-treated cells, blue – replicative senescence, gray – input signal

Out of 12 analyzed H3K9me3 methyltransferases, only 5 exhibited significant, treatment-dependent changes compared to young cells, and only a decrease in SUV39H1 and SUV39H2 was a characteristic of both types of senescence. SUV39H1 is a specific methyltransferase that, by trimethylating lysine 9 of histone 3, prompts its interaction with HP1 and, in consequence, chromatin condensation and stabilization [23]. Microarray results and subsequent qPCR reaction indicated a decrease in SUV39H1 gene transcript in both types of senescence (Fig. 4A). The observed decrease was reflected in protein levels (Fig. 4B). In addition, immunocytochemical staining showed a change in the distribution of the enzyme in the nucleus (Fig. 4C). Young cells had evenly and intensely stained nuclei with sporadic-single foci of SUV39H1. In RS and curcumin-treated cells, a decrease in fluorescence intensity was observed, and the occurrence of foci was negligible. However, in doxorubicin-treated cells, in addition to a general decrease in signal intensity, the formation of the highest number of SUV39H1 foci in the nucleus was detected (Fig. 4C). In contrast to RS and doxorubicin-treated cells, cells treated with curcumin additionally showed a slight decrease in the MECOM gene expression (otherwise known as PRDM3; a protein responsible for lysine 9 monomethylation [24]), SETDB2 (responsible for lysine 9 trimethylation [25, 26]) and an increase in PRDM16 (also responsible for lysine 9 monomethylation [24]) (Additional file 6, HMT).

##### H3K27 HMTs:

The analysis of enzymes responsible for methylation of lysine 27 of histone H3 (H3K27), included in the PRC2 complex and PRC2.1 and PRC2.2 variants, was carried out at the gene expression level. We also studied if the possible changes in gene expression were related to the deposition of active (H3K4me3), or silenced chromatin (H3K9me3) markers in the promoter and gene coding regions. The results have shown that the expression of the majority of genes encoding the PCR2 complex proteins decreased during senescence, which was probably responsible for the deregulation of the entire complex (Additional file 6). A crucial role in H3K27me3 trimethylation could be played by EZH1 and EZH2 [27]. EZH1 is often found in non-proliferating cells, in contrast to EZH2, which is characteristic of dividing cells [26]. The EZH1 protein has lower catalytic activity than EZH2, but is crucial for maintaining chromatin in a condensed form [28]. The level of the EZH2 gene transcript dropped in both types of senescence (Fig. 4E). Interestingly, there was an increase in EZH1 expression in all types of senescence (Fig. 4D). It cannot be ruled out that the increased expression of EZH1 maintains a constant level of H3K27me3. The elevated levels of EZH1 could be due to epigenetic regulation of the EZH1 gene. The ChIP-seq examination of the TSS region of *EZH1* showed enrichment in H3K4me3 in all experimental variants; however, only in control cells significant enrichment of repressing H3K9me3 was noted (Fig. 4G). Perhaps the inhibition or reduced level of expression of *EZH1* in CTRL occurs precisely by the presence of this marker right next to the TSS. It is possible that this results in local chromatin condensation and, thus, silencing of the gene expression. Similarly, regardless of the senescence type, a significant decline in EED protein (Fig. 4F) (not only in cells treated with curcumin, as the microarray results indicated – not shown) and gene encoding RBBP4 was observed [Additional file 6, HMT]. Lower levels of RBBP7 were noted only in DOX and RS cells. A decrease in SUZ12 was seen in RS cells. SUZ12, in cooperation with the histone chaperone proteins RBBP4/7, stabilizes the entire PRC2 complex by interacting with the N-terminal tail of histone H3 [29].

##### H3K4 HMTs:

The expression of genes encoding lysine 4 methyltransferase (H3K4) slightly decreases only in SETD1A (RS), SETD1B (DOX and RS) and WDR5 (RS). Exceptionally, a slight increase in expression occurs in the SMYD3 gene in cells treated with curcumin (Additional file 6, HMT).

#### Histone H3 demethylases (HDMs)

The transcriptomic profile of histone demethylases in all types of senescent cells was comparable with young cells and showed only slight, no significant changes (slightly above 1.3 threshold) in both directions (an increase or decrease in HDM mRNAs). It suggests that these enzymes may not play a key role in cellular senescence (Additional file 6, HDM). However, we did not analyze the activity of HDMs.

#### Histone H3 acetyltransferases (HATs)

The levels of 16 proteins involved in histone H3 acetylation were altered during senescence. A decrease in the transcripts of genes encoding most acetyltransferases was observed, except for three whose expression increased and depended on the type of senescence (Additional file 6, HAT).

#### Histone H3 deacetylases (HDACs)

**Fig. 5:**
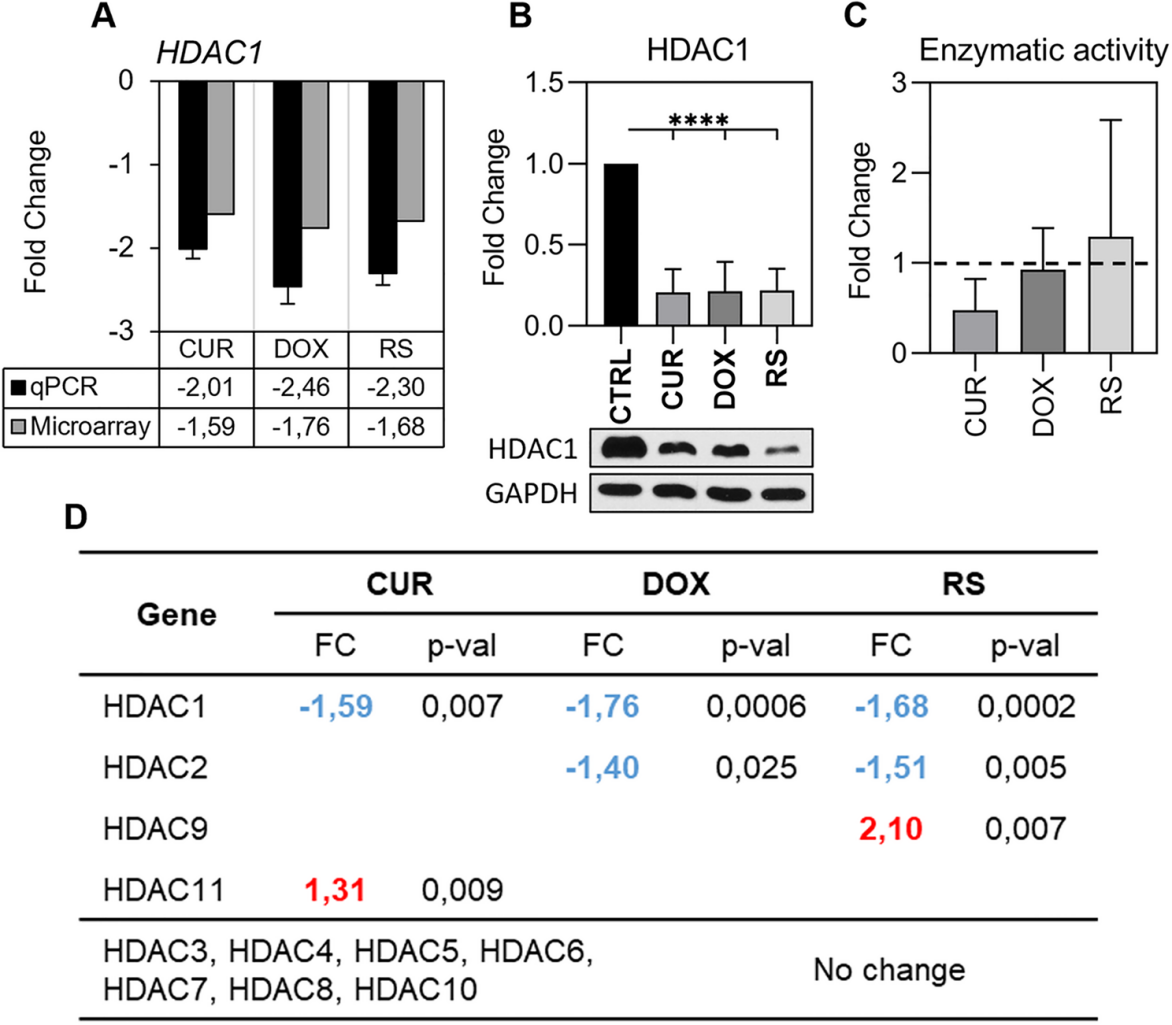
Analysis of selected histone deacetylases mRNAs, protein levels and activity in senescent cells. (**A**) Changes in HDAC1 mRNA level were measured using microarray (gray) and qPCR (black). (**B**) HDAC1 protein levels were determined by Western blotting. Densitometry results were prepared from three replicates, and a representative image is shown. Statistical analysis was performed using one-way ANOVA: *p* < 0.0001 (****). (**C**) Changes in enzymatic activity of a set of deacetylases (HDAC1, HDAC2, HDAC3, HDAC6, HDAC8, HDAC10 and HDAC11). (**D**) List of genes encoding histone deacetylases and the direction of changes observed during senescence (based on microarrays). The mRNA level is marked as the relative fold change in expression (FC) with the direction of change marked in color. Red indicates an increase and blue a decrease

During senescence, a decrease in *HDAC1* expression, which translated into protein level was detected (Fig. 5A and B). This decline was senescence-type independent. HDAC1 is a fairly well-studied protein that removes the acetyl group from lysine 9 of histone H3 [30]. However, this enzyme lacks DNA recognition and binding domain, so to properly perform its function, it must form a complex with, for example, a transcription factor or other chromatin-modifying proteins [31]. One such protein is SUV39H1 (the levels of which decrease in senescent cells), and the interaction of SUV39H1 and HDAC1 leads to chromatin condensation and gene silencing [32, 33]. The activity of a set of deacetylases, which includes HDAC1 and six other enzymes (HDAC2, HDAC3, HDAC6, HDAC8, HDAC10 and HDAC11) showed a subtle increase only in RS (no statistical significance) (Fig. 5C). On the other hand, the activity was almost halved in curcumin-treated cells due to the fact that curcumin is an inhibitor of deacetylases [34]. In contrast, in cells treated with doxorubicin deacetylase activity remained unchanged. The analysis of the other transcripts of genes encoding deacetylases showed that only three, apart from *HDAC1*, were altered. These were *HDAC2*, which decreased, and *HDAC11* (CUR) and *HDAC9* (RS), whose expression increased (Fig. 5D).

To summarize, the decrease in methylation of selected H3 lysine residues in senescent cells may be associated with a decrease in the expression of some histone methyltransferases (e.g., SUV39H1 and EED), deregulating the entire PRC2. Histone demethylases may play a less critical role. The observed alterations in HATs and HDACs were, in some cases, senescence-type dependent, but it seems crucial to analyze the involvement of particular enzymes to decipher their role in this process.

### HP1α level is reduced during senescence but shows senescence-type-dependent specificity

**Fig. 6:**
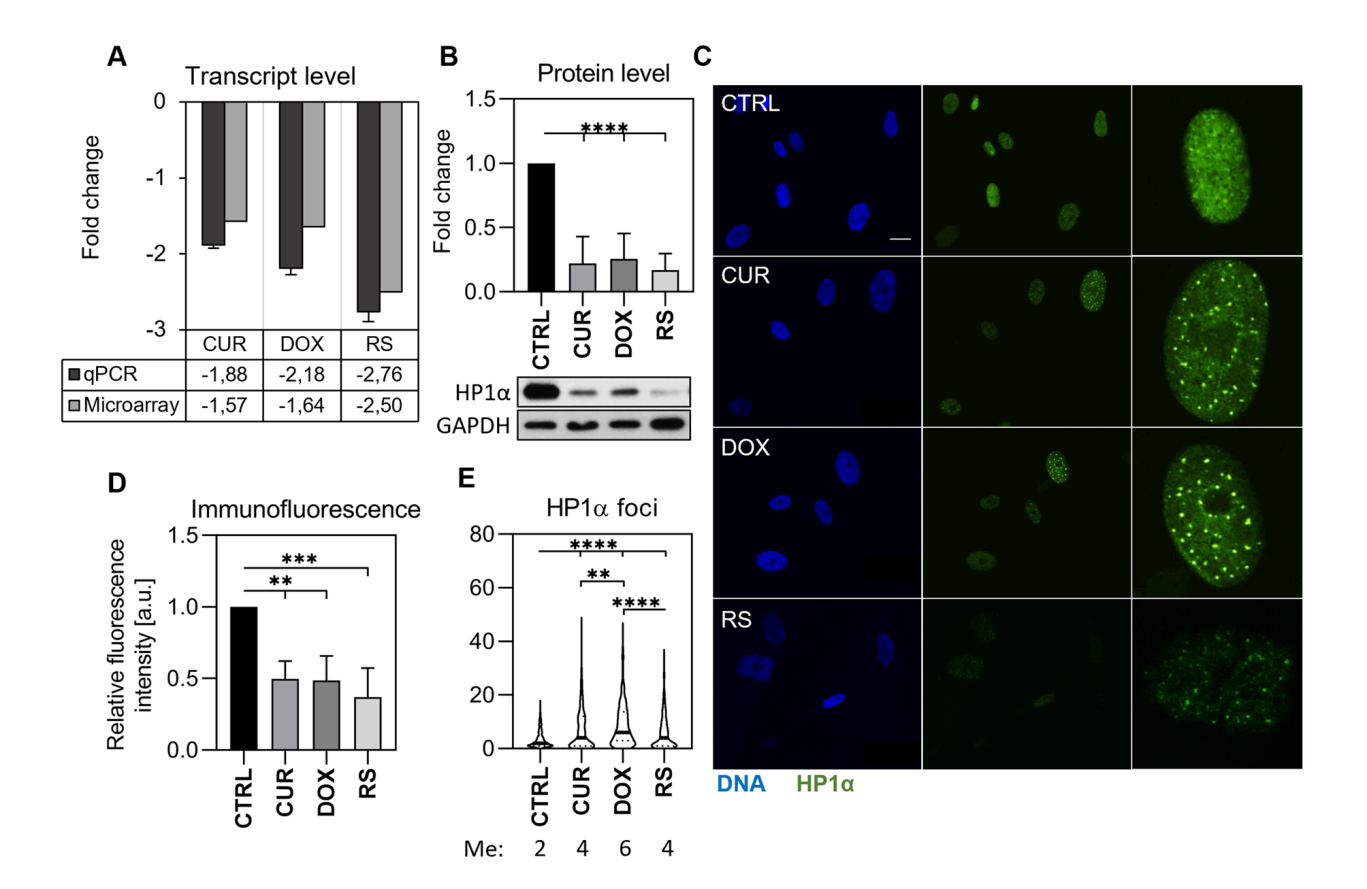
Changes in HP1α gene and protein expression accompanying different types of senescence. (**A**) mRNA level was determined by microarray (gray) and qPCR (black). (**B**) Densitometry results and representative images were obtained by Western blotting (*n* = 3). (**C**) Representative images of HP1α distribution in the nucleus (*n* = 3). Scale 10 μm. The far-right pictures show a zoom-in image of selected nuclei (**D**) Changes in the mean intensity of HP1α immunofluorescence staining in the nucleus (*n* = 3). (**E**) Graph showing the distribution of protein clusters (*n* = 4). The middle line marks the median, the exact value of which is given below the graph (Me). The dashed line marks the interquartile range. Statistical analysis was performed using one-way ANOVA: *p* < 0,05 (*), *p* < 0,01 (**), *p* < 0,001 (***), *p* < 0,0001 (****)

The HP1α protein attaches to trimethylated lysine 9 of histone H3 and is involved in forming transcriptionally inactive heterochromatin [35]. Our study has shown that the expression of HP1α decreases in all analyzed types of senescence (Fig. 6A and B). Immunocytochemical analysis of cell nuclei selected based on their surface area [manuscript in preparation] showed a decrease in the average fluorescence intensity of HP1α (per nuclear area) of senescent cells (Fig. 6D). It was accompanied by altered distribution of this protein in PS (Fig. 6C). In the nuclei of young control cells, few clusters of HP1α appeared (median is 2) (Fig. 6E), and the relatively intense fluorescence signal was mostly evenly distributed. In senescent cells, a decrease in fluorescence intensity and increased foci number were observed; however, the foci were less pronounced in RS than in PS. The highest number of HP1α foci was recorded in cells treated with doxorubicin.

**Fig. 7:**
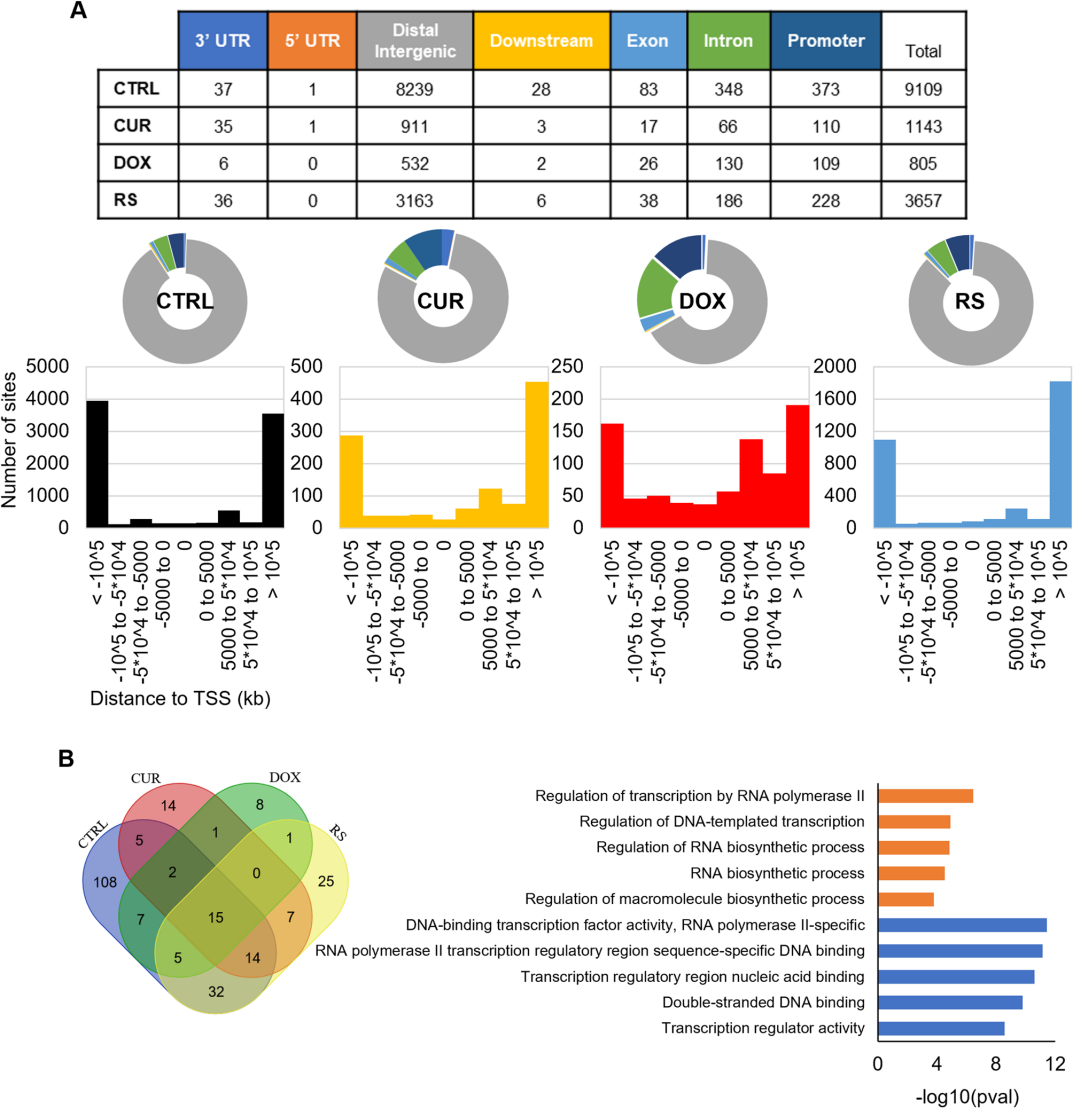
Distribution of HP1α in the genome of young and senescent VSMCs and functional analysis of sites of HP1α and H3K9me3 co-occurrence. (**A**) Table summarizing enrichment of HP1α in given areas of the genome for each experimental variant. The middle graphs show the percentage of reads of each area of the genome, and the panel below shows the distribution of enrichment sites around the transcription start site (TSS). The following labels were used: black - CTRL, yellow - CUR, red - DOX, blue – RS. (**B**) Venn diagram delineating intersecting sets containing common areas of enrichment of both HP1α and H3K9me3. The graph on the right shows functional analysis of the indicated HP1α and H3K9me3 co-occurrence sites characteristic only of young cells. The diagram lists terms for biological processes (orange) and molecular functions (blue). A detailed list of genes from all intersections is presented in Additional file 7

Moreover, the HP1α enrichment sites in the genome were investigated. As HP1α interacts with H3K9me3 and the level of both proteins decreases during senescence, we analyzed the co-occurrence of HP1α sites with H3K9me3. In light of previous reports that HP1a (an HP1α homolog) in *Drosophila melanogaster* may interact with chromatin independently of H3K9me3 [36], we decided to investigate the potential binding of HP1α to chromatin independently of H3K9me3 (using the ChIP-seq method) (Fig. 7).

The analysis of the enrichment sites of HP1α showed that the total number of reads was significantly lower than for H3K9me3. Moreover, the fewest number of reads was obtained for DOX-treated cells (Fig. 7). Similarly to H3K9me3, the highest number of HP1α reads was recorded in distal intergenic areas, and the percentage differed between variants. The DOX variant appears to have the strongest rearrangement of HP1α enrichment in the genome as the percentage of reads at distal intergenic sites decreased (Fig. 7A). The HP1α distribution around the TSS showed lower reads in each variant, with a significant proportion located in more distant regions of the genes. As the similarities in the distribution of reads between HP1α and H3K9me3 were detected, an analysis of an intersecting set of concurrent sites was illustrated by a Venn diagram (Fig. 7B left). As can be noted, the most significant number of such interaction sites occurred in young cells. Therefore, only genes identified in young cells were subjected to functional enrichment analysis. The concurrent HP1α and H3K9me3 sites were associated with the regulation of transcription and RNA biosynthesis, and the genes involved in these processes belong mainly to the ZNF family of transcription factors. Attempts were made to identify sites of HP1α binding with chromatin not involving H3K9me3. The analysis did not reveal many sites; no genes were common for senescence types.

### The transcriptomic profile differs in premature and replicative senescence

**Fig. 8:**
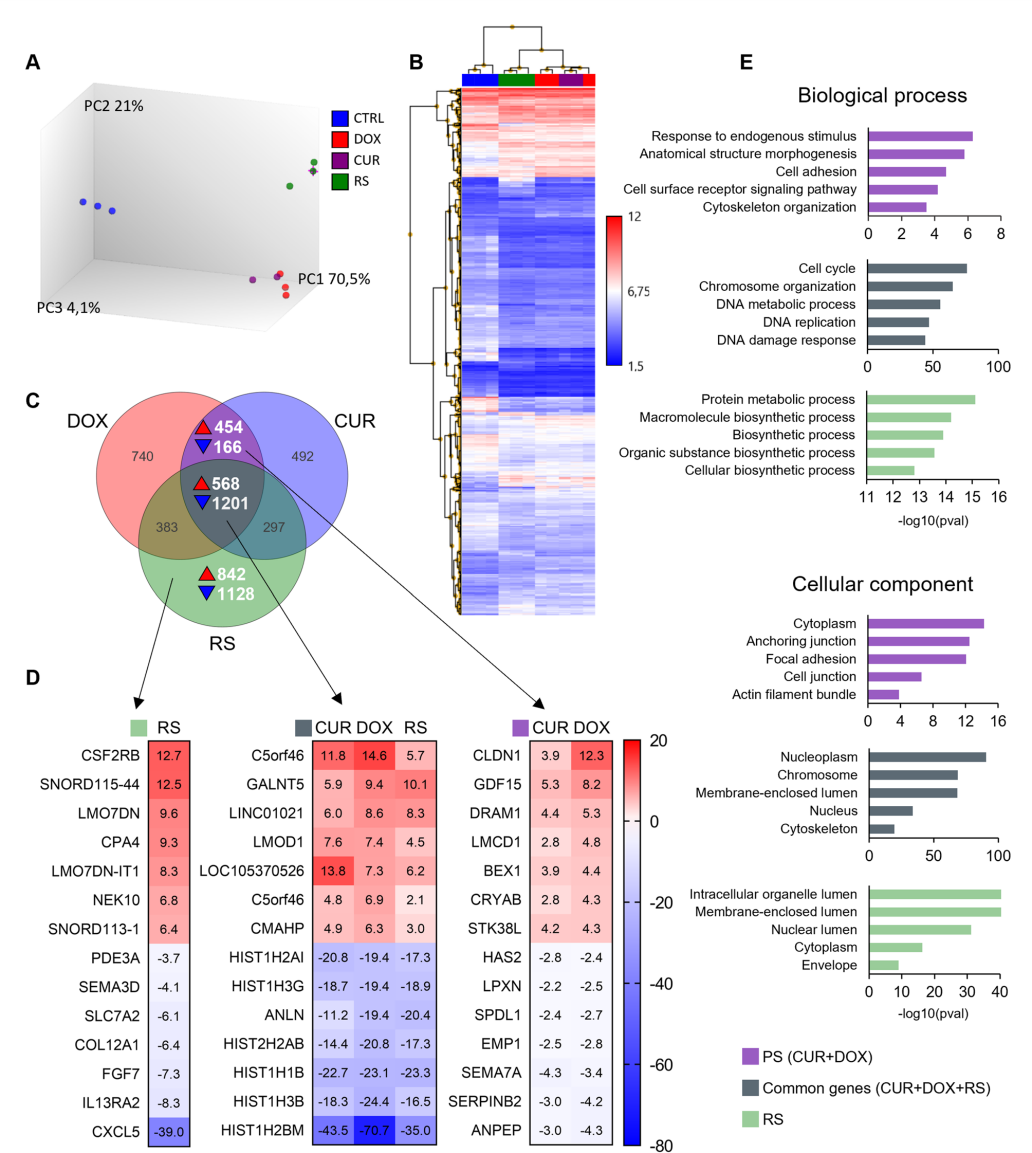
Summary of changes in transcriptomic profile in young and senescent cells. (**A**) Principal component (PC) analysis shows differences between RS (green dots) and PS (DOX cells - red, CUR cells - purple) and between the two types of senescent cells and young cells (blue dots). Image prepared with TAC software. (**B**) DEG hierarchical clustering with log2 heat map of the signal recorded for each gene. Image prepared with TAC software. (**C**) Venn diagram showing the intersections of DEGs in each experimental variant relative to young cells, including the number of genes in each subset. For each type of senescent cells: PS (DOX and CUR), RS, and PS + RS the number of genes with increased (red triangle) and decreased (blue triangle) expression is indicated. (**D**) A list of the 7 most upregulated and the 7 most downregulated genes in each set (PS - DOX and CUR, RS and common for PS and RS). On the right is a heat map showing Fold Change. (**E**) Functional analysis of genes specific to the studied compartments (PS, RS and common for PS and RS). The graphs show biological processes and include the location in the cell where the gene product has a specific function (cellular component). The analysis was performed using the g: Profiler tool, which relied on the Gene Ontology database. List of genes characteristic exclusively for senescence induced by DOX or CUR is presented in Additional file 8

Functional enrichment analysis of RS and PS-specific genes was performed to identify changes occurring in senescent VSMCs based on the results obtained from microarrays (Fig. 8). Principal component analysis (PCA) showed significant differences in the transcriptome between young (blue) and RS cells (green), young and PS cells (red - DOX, purple - CUR), and between the two types of senescent cells (PS and RS) (Fig. 8A). The results support the existence of differences between stress-induced and replicative senescence. Differentially expressed genes (DEGs), specific to each senescence variant, are also shown in a heatmap form and as hierarchical clustering of both genes and variants (Fig. 8B). The colors on the heatmap represent the recorded signal (log2), where blue indicates the lowest and red is the highest signal. The clustering for experimental variants provided the same conclusions as the PCA analysis. Therefore, it was decided to analyze gene expression changes common to all senescence variants. In the Venn diagram, the intersecting sets of DEGs were marked accordingly, and their expression was normalized to control cells (Fig. 8C). Seven genes with the highest and lowest expression for each set are shown (Fig. 8D). The functional enrichment analysis was then preformed for each set of genes in the studied group (PS, RS, genes common to PS + RS) using the Gene Ontology database, and the results are presented for biological processes and cellular components (Fig. 8E). List of genes characteristic exclusively for senescence induced by DOX or CUR is presented in Additional file 8.

The results showed that genes with altered expression in all types of senescence (referred to genes common to all types of senescence), encode proteins located mainly in the nucleus, where they are involved in cell cycle control, chromosome organization, replication and DNA repair. Significant differences in gene functions can be seen between RS and PS. In the case of RS, gene products are associated with maintaining metabolic processes and protein synthesis and are most often found inside various cell organelles, including the nucleus and the cytoplasm. Cells treated with doxorubicin and curcumin (PS) show changes in the expression of genes related to response to external stimuli. Interestingly, these genes are also involved in cell adhesion and cytoskeletal reorganization. The proteins encoded by these genes localize in the cytoplasm and are associated with actin filaments and cell adhesion sites. Changes in their expression could affect cell migration, as we have previously observed for PS cells (in preparation).

### Changes in histone modifications and HP1α distribution in VSMCs derived from atherosclerotic plaques (ex vivo) reflect alterations detected in in vitro senescence

**Fig. 9:**
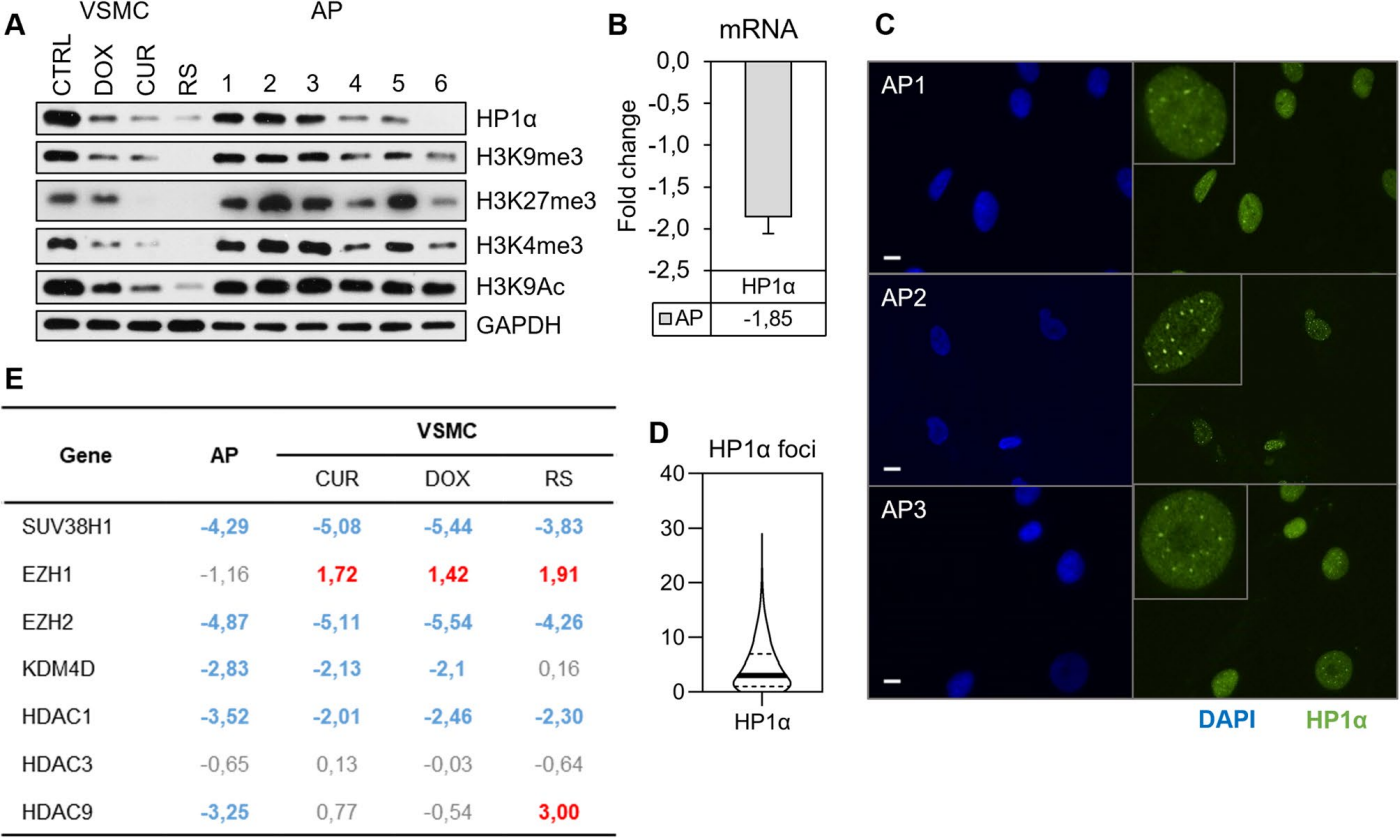
Changes in the level and localization of histone modifications, HP1α and expression of histone-modifying enzymes in cells derived from atherosclerotic plaques (AP). (**A**) Examples of changes in the level of HP1α, H3K9me3, H3K27me3 (heterochromatin markers) and H3K4me3 and H3K9Ac (euchromatin markers) obtained by Western blotting from cells derived from 6 donors (right side of the blot) compared to VSMCs in vitro (left side of the blot). (**B**) HP1α mRNA level measured by qPCR and presented as a fold change relative to young VSMCs from in vitro culture, *n* = 3 (**C**) Representative pictures of HP1α analyzed by immunofluorescence of cells derived from three donors; 10 μm scale (**D**) Graph showing the HP1α foci number in AP cells from 3 donors. The middle line marks the median, the exact value of which is marked on the graph (Me = 4). The dashed line marks the interquartile range. (**E**) The Expression of histone modification enzymes was analyzed by qPCR and compared to that of VSMCs in vitro

Analysis of senescence markers showed that atherosclerotic plaques (AP) consist of a heterogeneous population of young and senescent cells. Due to different proportions of senescent cells, the expression of senescence markers is strongly donor-dependent. Cells derived from atherosclerotic plaques may preserve young cell morphology, but, at the same time, decreased levels of LMNB1 (lamin B1) and HMGB1 (High mobility group box 1 protein), recognized senescence markers, were detected in whole cell extracts (Additional file 1). About 60% of the cells showed increased SA-β-gal activity, but approximately 60% could still replicate DNA (Additional file 1). The level of histone H3 modifications was strongly donor-dependent and mostly resembled that in young VSMCs cultured in vitro (Fig. 9A). For comparison, we have shown VSMCs senesce in vitro. We normalized all H3 modifications in VSMCs and AP cells to GAPDH level because, in this manner, it is more visible that the level of studied modifications did not change spectacularly in AP cells. As we have shown for VSMCs, in the case of H3K9me3 and H3K4me3 normalization to H3 and GAPDH gave the same results; however, in the case of H3K27me and H3K9Ac their relative level decreased if GAPDH served as a loading control. In AP cells, the level of all studied H3 modifications remained relatively high. The HP1α level was also donor-dependent, and the direction of HP1α changes in individual donors correlated with the heterochromatin marker H3K9me3 (Fig. 9A). mRNA analysis demonstrated a drop in HP1α gene expression in AP cells (Fig. 9B). The immunostaining analysis showed that the average fluorescence intensity of HP1α protein decreased with increasing nucleus area (Fig. 9C). Similarly to the in vitro senescence of VSMCs, there was an increase in the number of HP1α foci in the large nuclei of senescent cells (selected based on the area > 200 μm) compared to young ones (selected based on the area < 200 μm), in which foci appeared in smaller numbers (Fig. 9C and D). Immunocytochemical staining showed that the fluorescence signal intensity of the tested markers was highly variable and donor-dependent, which is consistent with observations at the protein level.

We applied qPCR to measure the level of genes encoding selected histone-modifying enzymes (Fig. 9E). The results are presented as a fold change relative to young VSMCs and compared to senescent VSMCs. The expression of the majority of genes decreased (SUV39H1, EZH2, KDM4D, HDAC1) or remained unchanged (HDAC3), which was consistent with those obtained for VSMCs. Surprisingly, in contrast to VSMCs, there was no significant change in EZH1 expression, and the expression of HDAC9 decreased. This might be a result of a more heterogenous population of AP cells. Nevertheless, the trend in enzyme expression mostly resembled this observed in the in vitro model.

### The alterations observed in VSMCs are universal, as they also occur in fibroblasts subjected to different types of senescence

We performed selected analyses on human fibroblasts to recognise whether senescence-associated alterations in H3 modifications and HP1α in VSMCs are cell type-specific or universal. Senescence was induced in a manner similar to that of VSMCs. The percentage of senescent and non-proliferating cells was evaluated, and the expression profile of typical senescence markers, LMNB1 and HMGB1, was analysed (Additional file 2). However, unlike in the case of VSMCs, young fibroblasts that served as control cells were collected 48 h after seeding since preliminary experiments showed that, at this time point, they were proliferating most intensely and were, therefore most suitable for studying differences with respect to senescent cells. In some analyses, we also show results for cells collected 24 h after seeding for comparison. According to the literature, in early passages between 1 and 20, the time for cell division is 30–40 h [37, 38].

**Fig. 10:**
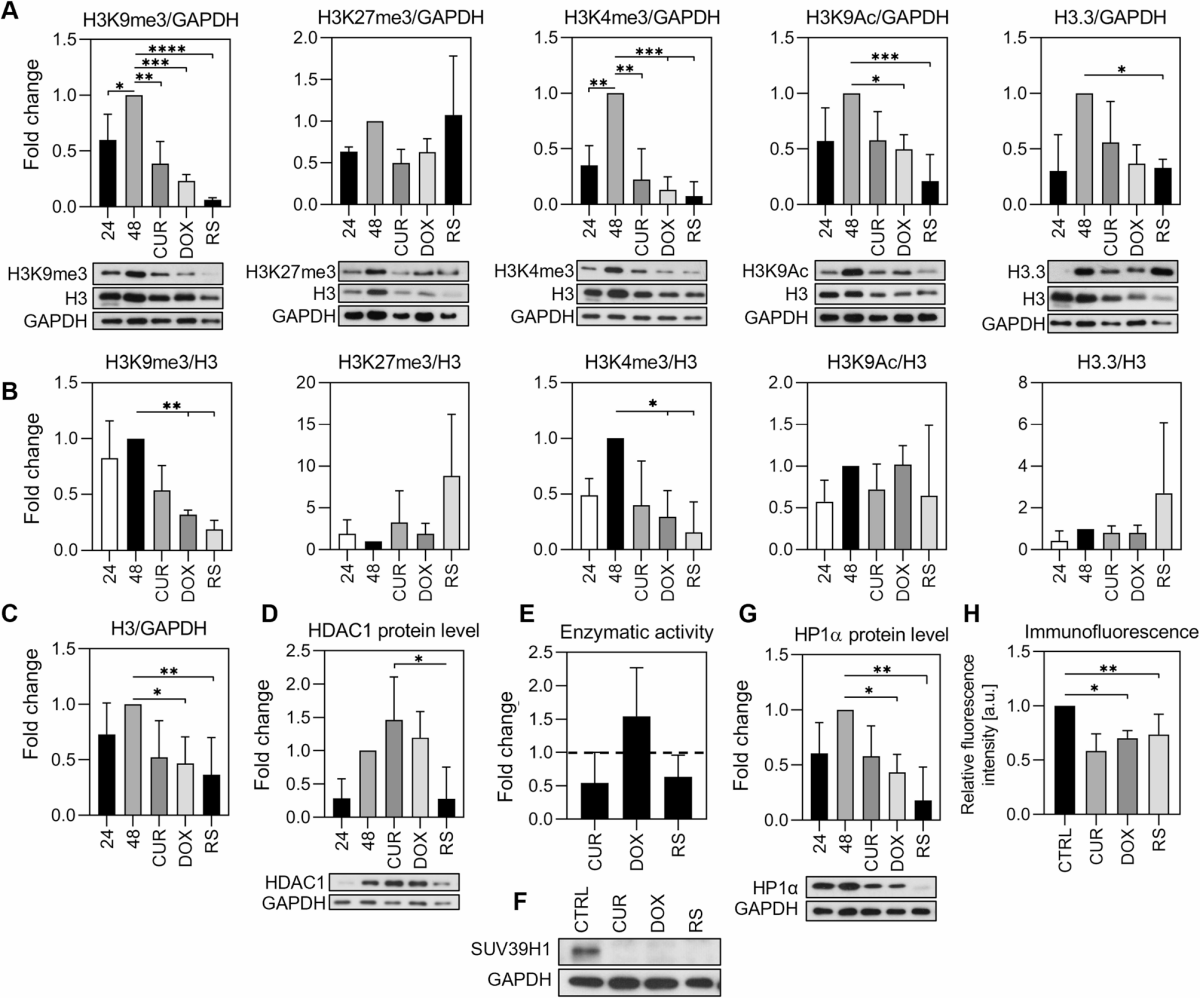
Changes in the levels of histone H3, its variant H3.3, histone H3 modifications, HP1α and enzymes responsible for histone H3 modifications in young and senescent fibroblasts. (**A**) Changes in histone modifications (H3K9me3, H3K27me3, H3K4me3, H3K9Ac) and H3.3 variant normalized to GAPDH. (**B**) and to H3. (**C**) Changes in H3 protein level normalized to GAPDH. **D** Changes in HDAC1 protein level. (**E**) Enzymatic activity of histone deacetylases (HDAC1, HDAC2, HDAC3, HDAC6, HDAC8, HDAC10 and HDAC11) relative to control (dashed line). (**F**) Changes in SUV39H1 protein level. (**G**) Analysis of HP1α protein level in fibroblasts measured by Western blotting. (**H**) Measurement of HP1α fluorescence intensity. All assays were performed in 3 biological replicates and in most cases presented as mean ± SD. **A** - **D**, **F**, **G** - densitometric analysis and representative image from Western blotting. Proteins were normalized do GAPDH or H3 and presented as fold change relative to young, 48 h control cells. Statistical analysis was performed relative to 48 h control using one-way ANOVA: *p* < 0.05 (*), *p* < 0.01 (**), *p* < 0.001 (***), *p* < 0.0001 (****)

In fibroblasts, a significant decrease in H3K9me3 in both senescence types was observed (Fig. 10A), which reflects the alterations observed in VSMCs (Fig. 1A). The H3K27me3 level tended to increase, without statistical significance, if compared to the H3. Diminished H3K27me3 level was noticed in PS when normalized to GAPDH. Euchromatin markers showed a similar trend to those observed in VSMCs - that is a noticeable decrease in H3K4me3 in both types of senescence (normalized to both H3 and GAPDH), no significant alterations in H3K9Ac when compared to H3 and a reduction if normalized to GAPDH. Similarly to VSMCs, neither H3K4me3 nor H3K9Ac diversification between PS and RS was detected. In both senescence types, there was also a decrease in histone H3 itself (Fig. 10B) and its variant H3.3 (irrespective whether normalized to H3 or GAPDH, statistically significant only with GAPDH) (Fig. 10C). In conclusion, when the level of histone modifications in senescent fibroblasts was compared to proliferating ones (control 48 h), changes in the level of H3K9me3, H3K4me3, H3K9Ac and H3K27me3 resembled those observed in VSMCs. Thus, a reduced level of H3K9me3 and H3K4me3 appears to be a universal senescence marker. In addition, there was a senescence-related overall decrease in histone H3, which could result in nucleosome loss and chromatin loosening. Analysis of the level of H3 modifications in control cells as a function of the time of culture showed low levels of all tested modifications 24 h after seeding. This is related to the delayed entry of cells into the cell cycle (Additional file 2). Immunocytochemical analysis showed a decrease in the fluorescence intensity and significant similarities in the distribution of all markers that accompanied the senescence of VSMCs (Fig. 1B).

Analysis of enzymes regulating chromatin structure by modifying histone H3 showed inhibition of SUV39H1 expression (Fig. 10D) in fibroblasts regardless of the senescence type. Similar results were obtained in VSMCs. In contrast to VSMCs, in fibroblasts the level of HDAC1 increased in PS and decreased in RS cells (compared to the 48-hour control) (Fig. 10E). The highest increase of HDAC1 was recorded in cells treated with curcumin, which was, however, not reflected by protein acetylation status (activity of the deacetylase set, Fig. 10F). In contrast, the highest activity of deacetylases was observed in doxorubicin-treated cells.

HP1α synthesis was reduced in all types of senescence, with the most spectacular drop observed during RS (Fig. 10G). This was also confirmed by immunocytochemical staining (Fig. 10H). To eliminate population heterogeneity, a similar criterion to cell nuclei size selection as in VSMCs (in vivo and ex vivo) was applied to fibroblasts. Unlike VSMCs, no accumulation of HP1α foci in the nucleus was observed during fibroblast senescence (not shown).

## Discussion

Many studies have shown that the presence and accumulation of senescent cells in tissues and organs significantly impact their function and are closely related to organismal aging. Senescence of VSMCs is involved in atherosclerosis progression and plays a critical role in the formation and stabilization of atherosclerotic plaques [39]. We have earlier described in some detail various changes accompanying replicative and premature senescence induced by doxorubicin (DOX) and curcumin (CUR) in this cell type [6–8]. Doxorubicin causes double-strand DNA damage, reflected in altered chromatin structure and gene expression. This drug is used in cancer therapy, where its cardiotoxic effect [40] and contribution to the senescence of cardiac progenitor cells and cardiomyocytes were documented [41, 42]. Curcumin induces DNA damage-independent senescence even though it activates the DNA Damage Response (DDR) pathway. In this study, we aimed to characterize changes in selected proteins that can affect chromatin structure and identify features that differentiate these two types of senescence - RS and PS. It seems to be especially important since both types of senescence occur concomitantly in the organism. The new senescence markers may allow for identifying conditions responsible for the induction of accelerated aging and, in a further perspective, for selecting strategies to mitigate or even prevent the adverse effects of aging. Specific markers that could discriminate premature from replicative senescence are unknown. Given that histone H3 modifications regulate chromatin structure and gene expression, and that some alterations have already been documented in senescent cells [43], we chose them to characterize VSMC senescence and to distinguish its various types. The role of HP1α in senescence progression is not yet understood.

Even though PS and RS of VSMCs have been previously characterized and fibroblasts are the best-studied cell type in terms of senescence, changes in the nucleus architecture and chromatin structure during these two types of senescence remain poorly understood. We observed that some changes associated with senescence were universal (i.e. followed a similar pattern in VSMC and fibroblasts, e.g., decrease in H3K9me3, H3K4me3, HP1a and SUV39H1). Interestingly, differences between RS and PS were detected, and some were cell-type dependent. In the population of VSMCs derived from atherosclerotic plaques, the presence of senescent cells has been proven [44] and they were characterized by higher SA-β-gal activity, higher levels of p21 and p16 proteins and increased DNA damage, recognized by the appearance of γH2AX foci. The senescence-associated nuclear alterations in ex vivo VSMCs were not elucidated in detail. However, we have noted that the expression of senescence markers in plaque-derived cells was strongly donor-dependent (due to VSMC heterogeneity), and therefore, the results, in some aspects, were difficult to interpret. This discrepancy in the results may be due to the fact that the characteristics of plaques from which the VSMCs were isolated were diverse - ranging from very unstable plaques, which led to ulceration, rupture and ultimately to stroke, to much more stable plaques, which caused significant hemodynamic stenoses but did not cause neurological symptoms. Referring to the dependence on the age of donors, comorbidities, the degree of calcification or plaque features requires analyzing a suitably large material (from numerous donors divided into groups depending on the previously mentioned features).

Senescence-type-dependent (PS and RS) alterations in the transcriptomic profile of VSMCs were documented and the principal component analysis showed a high resemblance between cells induced to senesce with doxorubicin and curcumin (Fig. 8A). PS and RS cells differed strongly from young ones. Functional enrichment analysis yielded three groups of genes: common to all types of senescence, changing expression only in PS (regardless of senescence-inducing stimulus) and characteristic only of RS. According to the Gene Ontology database, genes that change expression in all types of senescence are involved, among others, in cell cycle regulation, DNA replication or DNA damage response, which is consistent with previous reports and reflects the nature of the senescence process. In contrast, interesting differences were observed between PS and RS. Genes that change expression in PS cells are involved in response to an external stimulus, cell adhesion or cytoskeletal organization. Proteins encoded by these genes are mostly located in the cytoplasm, anchoring junctions, cell-cell junctions or actin bundles. This suggests that cells subjected to premature senescence may be able to migrate. Such observation has indeed been made by us (manuscript in preparation), although literature data provide conflicting information on this subject [45, 46].

Examination of the transcriptomic profile in VSMCs subjected to doxorubicin-induced senescence was performed by Uryga’s team [47]. VSMCs were derived from elderly donors and the cells treated with doxorubicin were compared to proliferating untreated cells. The separate analysis of RS versus proliferating cells or transcriptomic comparison between these two types of senescence (DOX and RS) was not clearly reported in the study. Genes that were activated during DOX-induced senescence regulate SASP (senescence-associated secretory phenotype) and DNA repair; in turn, silenced genes were associated with the cell cycle and division, and maintaining the telomere structure. When functional analysis of the results on doxorubicin treated cells, obtained in this study, was carried out using the REACTOME database, inhibition of similar pathways was observed, while processes related to SASP were predominantly activated. Analysis of RS in VSMCs performed by us indicated that genes specific to this type of senescence were mainly responsible for regulating metabolic processes and the biosynthesis of various cellular molecules. Since there is no clear functional analysis of transcriptome in RS, our results were confronted with proteomics data [48]. Functional enrichment analysis of proteins suggests deregulation of processes related to RNA metabolism (as indicated by downregulated secretion of proteins), which we did not observe on the transcript level. Our data are the first that thoroughly compare gene expression changes occurring in replicative and premature – induced by DNA-damage dependent and independent manner – senescence. Based on the microarray results, a significant senescence-associated decrease, as high as 70-fold, was noted in the transcription of genes clusters encoding various histones, with exemptions of several not changing (e.g. HIST1H2BC – data not shown) or slightly increasing in SIPS or RS (e.g. HIST1H2BD and HIST1H1T respectively – data not shown). This, to some extent, contradicts the results obtained by Uryga’s team, as their data showed a significant increase in the expression of selected histone encoding genes in doxorubicin-induced senescence [47]. The discrepancies observed between our findings and those reported by Uryga et al. may, in part, stem from differences in the source and donor age of the VSMCs used in each study. Uryga’s team utilized primary aortic VSMCs derived from elderly donors (mean age − 65 years, both male and female), which were proliferation-competent. In our experiments, cells were isolated from adult male donors (aged 30), and to obtain RS, the cells were passaged until they lost their proliferative potential. This age gap is particularly relevant given the well-documented shifts in the epigenetic landscape that occur throughout the human lifespan, including changes in DNA methylation patterns and histone modifications [49–51]. Furthermore, the senescence induction protocols used in the two studies also differed. Uryga et al. employed a SIPS model involving a 24-hour doxorubicin treatment followed by a 21-day recovery period, while our approach involved continuous exposure to doxorubicin for seven days. This variation in protocol could influence the cellular senescence phenotype, as it was shown that acute versus “established” SIPS – defined by longer recovery periods – exhibit differences in the expression of senescence markers such as p16 and p21 [47]. These factors together may contribute to the observed differences in gene expression profiles between the studies. Moreover, we examined histone H3 at the protein level. As expected (considering the microarray results), there was a decrease in H3 levels in all senescence variants tested, both in VSMCs and fibroblasts, which is a characteristic feature of senescence [52]. As the senescent cells are arrested in the cell cycle, we additionally examined H3.3 variant. Its incorporation is independent of cell division and plays a role in chromatin organization and function, regulates gene expression and genome integrity. It is involved in both transcriptional activation and repression, depending on the genomic location and the specific histone chaperones [20, 21]. We have noticed cell-type-dependent differences in the H3.3 level. In fibroblast, H3.3 seemed to decrease; however, in VSMCs, an elevating tendency was observed for RS. These observations partially contradict literature data, as many studies confirm an increase in H3.3. in senescent cells [53].

One of the typical features of cellular senescence is a significant increase in the nucleus area and chromatin relaxation (deheterochromatinization) [54–56]. We analyzed the senescence-associated changes responsible for chromatin structure regulation in different cell and senescence types by studying selected markers characteristic of hetero- and euchromatin and histone H3 modifying enzymes. We confirmed that cellular senescence was associated with a decrease in H3K9me3, however, we showed that the extent of decline depended on the cell and senescence types. In VSMCs, a decrease in H3K9me3 was observed, with the most significant being noted in RS cells. A senescent-specific decrease in the SUV39H1 methyltransferase was most likely responsible for lower lysine 9 methylation. Moreover, in DOX-treated cells, multiple SUV39H1 foci were observed. This could be associated with DNA double-strand breaks, occurring mainly in cells treated with doxorubicin, and link the altered location of SUV39H1 with DNA break repair.

Increased expression of the gene encoding demethylase KDM4B (Additional file 6), which has the highest affinity for H3K9me3 [57], may also be responsible for the decrease in H3K9 trimethylation in VSMCs [58]. Analysis of the distribution of H3K9me3 (ChIP-seq) indicated a decrease in this modification precisely in the close vicinity of centromeres and telomeres, where constitutive heterochromatin is found (analysis using IGV Additional file 3). We observed a decreased gene expression of enzymes responsible for H3K9 trimethylation, while there was no change in the enzymes responsible for mono- and demethylation. It can be assumed that VSMC senescence is accompanied mostly by a decrease in H3K9me3 and relaxation of constitutive chromatin. Similar observations have been made in fibroblasts of the accelerated aging model (Cockayne syndrome) [59]. H3K27me3 (facultative heterochromatin marker) did not alter in the studied cell types. A decrease in H3K27me3 and H3K9me3 was observed in advanced stages of the atherosclerotic plaque, with a concomitant increase in H3K9 and H3K27 acetylation, which destabilizes the atherosclerotic plaque in patients [60]. This was not due to alterations in either EZH2 methyltransferase or JMJD3 demethylase expression, as their expression did not change significantly in atherosclerosis progression [61]. This contradicts our results to some extent since the expression of EZH2 and other proteins that form the PRC2 complex decreased in all types of senescence. The gene encoding a homolog of EZH2, the EZH1 protein, proved to be an exception; however, its role in VSMC senescence remains unclear. In hematopoietic cells, higher levels of EZH1 are associated with a decrease in H3K27 trimethylation with a concomitant increase in H3K27 monomethylation and H3K4 trimethylation. Moreover, it has been shown that EZH1 may also act independently of EED (enzymatic part of methyltransferase complex PRC2) [62, 63], whose transcript and protein levels decreased in our model. In VSMCs subjected to PS and RS, despite the probable insufficiency of the PRC2 complex, H3K27 trimethylation was not altered.

Changes in heterochromatin markers in both cell types (VSMCs and fibroblasts) suggest that the most substantial deheterochromatinization is mainly related to RS, while PS cells show an intermediate degree of chromatin packing. Results concerning atherosclerotic plaques showed donor-dependent changes in heterochromatin markers (probably due to different percentage of senescent cells).

Unexpectedly, VSMC senescence was associated with a decrease in H3K4me3 and unaltered level of H3K9Ac, which are active chromatin markers. This is inconsistent with previously published studies that have demonstrated an increase in H3K4me3 and H3K9Ac in between early and advanced stages of atherosclerosis [60]. This increase was associated with an increased number of senescent cells in the plaque. Increased acetylation of H3K9 has also been documented in RS [64]. Since our study has shown no changes in H3K9 acetylation and diminished H3K4me3 level, we studied the enzymes responsible for H3K4 methylation and H3K9 acetylation. We have shown that histone demethylases may be responsible for the decrease in H3K4 trimethylation since the expression of KDM5D and KDM5B genes increased in senescence (depending on its type Additional file 6). It cannot be ruled out that methyltransferases also contribute to the lower level of H3K4me3 in RS, since the expression of genes encoding three of them (SETD1A, SETD1B, WDR5) decreased (Additional file 6). Despite the overall decline in H3K4me3 levels in senescent cells, changes in this modification’s enrichment profile in the genome were insignificant. ChIP-seq analyses showed senescence-dependent reorganization of H3K4me3 with new sites appearing, depending on the type and the factor inducing senescence. In addition, it has been proven that promoters of many genes encoding proteins involved in the cell cycle or metabolic processes remained consistently enriched in H3K4me3 in all experimental variants. However, according to a comparative analysis of transcriptomic results, the presence of this activating modification did not necessarily correlate with gene expression. Some genes remained silenced despite the presence of H3K4me3 in the promoters (e.g., some of the E2F family protein genes) (Additional file 4). It can be concluded that perhaps other mechanisms are responsible for controlling expression of these genes. One of these may be the simultaneous presence of H3K27me3 on the promoters [65]. Such bivalently marked promoters are usually transcriptionally inactive or expressed at very low levels [66]. Another mechanism for regulating gene expression may be the binding of different transcription factors, which can be associated with wide or narrow peaks in the promoters. In addition, very often, there was a reduction in the peak height of H3K4me3 reads in senescence. Recent studies suggest that high H3K4me3 peaks may be related to the contractile phenotype of VSMCs controlled by the SMYD3 methyltransferase [67], but we did not observe changes in SMYD3 expression.

We checked the expression of genes encoding histone deacetylases, their activity and HDAC1 protein level. In senescent cells, we observed a decrease in the level of HDAC1 (responsible for lysine 9 deacetylation). Interestingly, in RS, there was an increase in the activity of HDAC1 and numerous other HDACs. Thus, higher activity may compensate for a decrease in HDAC1 at the protein level or other deacetylases could take over its functions. Unexpectedly, there was an increase in HDAC1 expression in senescent fibroblasts, and the highest activity was observed in cells treated with doxorubicin. Microarray results showed that mRNA of all sirtuins, the most widely described histone deacetylases involved in atherosclerosis progression, remained on the same level as in control cells. This is a rather surprising result in the context of previous studies showing significant changes in the protein level of selected sirtuins in PS and RS [7, 8, 68].

Finally, the involvement of HP1α in senescence has been investigated. This protein plays a significant role in maintaining the nuclear shape via association with the nuclear envelope and heterochromatin [69]. Existing data confirm that a decrease in HP1α is a characteristic feature of senescent cells in progeria patients (HGPS - Hutchinson-Gilford progeria syndrome, Werner syndrome) [70, 71]; however, the role of HP1α in RS and in senescence unrelated to DNA damage has not been clarified yet. We have shown that in VSMCs, gene expression and HP1α protein levels decreased significantly regardless of senescence type. Instead, characteristic HP1α foci were formed, most likely by liquid-liquid phase separation [72]. The highest number of foci was observed in cells treated with doxorubicin (an average of 6 foci per nucleus) and slightly fewer in cells treated with curcumin or subjected to RS (4 foci). Such concentration sites were not observed in fibroblasts.

Although HP1α interacts with H3K9me3, its silencing did not affect H3K9me3 levels, chromatin packing, or gene transcription [73]. We have studied the distribution of HP1α in the genome. The highest number of reads for HP1α was observed in intergenic regions, supporting a possible effect on chromatin condensation and regulation of gene expression. Similar observations on the presence of HP1α in intergenic regions were reported for embryonic kidney HEK293 cells, in which 73% of all reads originated from those loci [74]. In our experimental model, they accounted for 90% of reads. In HEK293 cells and in the VSMCs studied by us, an increase in HP1α coverage was mainly observed in genes encoding the ZNF family proteins (transcription factors). Genes encoding ZNF proteins were also abundant in H3K9me3, becoming the site of the most frequent co-occurrence of HP1α and H3K9me3. ZNF proteins are associated with the regulation of gene transcription and RNA biosynthesis, but their role in senescence remains unrecognized to date. Contrary to expectations, the ChIP-seq analysis did not provide information on the occurrence of HP1α in the genome independently of H3K9me3. Our study was the first attempt to characterize the shift in HP1α distribution in the genome of senescent VSMCs, and may provide a valuable basis for future studies.

### Concluding remarks

We characterized senescence type-dependent changes in chromatin-associated proteins and enzymes that influence the nucleus and chromatin structure in VSMCs. This study can serve as a starting point to search for factors that may be used to distinguish between premature and replicative senescence. We mapped the deposition of H3K4me3, H3K9me3 and HP1α in the genome of VSMCs subjected to PS and RS and described transcriptomic alterations that arise depending on the senescence-inducing stimulus. We compared the profile of H3 modifications and HP1α level and distribution characteristic for VSMCs with changes detected in fibroblasts and, to some extent, in VSMCs derived from atherosclerotic plaques. A phenomenon that seems to be universal regardless of cell type and senescence stimulus, is a significant decrease in H3K9me3, H3K4me3 and HP1α levels. In contrast, alterations detected in cells derived from atherosclerotic plaques were more variable and donor-dependent. Our study provides evidence that changes in the structure of the nucleus and chromatin, associated with a gradual loosening of chromatin, are universal for all senescence types. However, the highest extent of deheterochromatinization is a feature of cells undergoing RS. We can also conclude that certain similar alterations occur in senescent VSMCs ex vivo, although inter-individual differences usually obscure them. Our results clearly showed that differences existed not only between young and senescent cells but also between PS and RS ones. Only subtle differences between various PS types were observed, suggesting that different stressors activate the same cellular mechanisms/pathways.

## Electronic supplementary material

Below is the link to the electronic supplementary material.

Supplementary Material 1: Additional file 1 - Verification of senescence model in VSMCs in vitro and evaluation of senescence/proliferation state of VSMCs isolated from atherosclerotic plaque (*ex vivo*)

Supplementary Material 2: Additional file 2 - Control selection and verification of PS and RS efficacy in fibroblasts

Supplementary Material 3: Additional file 3 - The visual enrichment analysis in IGV software of H3K9me3 in the pericentromeric and subtelomeric regions of chromosome 7

Supplementary Material 4: Additional files 4 - Enrichment analysis of H3K4me3 in the promoter regions of E2F family protein-encoding genes

Supplementary Material 5: Additional files 5 - Primers sequence

Supplementary Material 6: Additional files 6 - Enzymes responsible for histone modifications

Supplementary Material 7: Additional files 7 - List of regions enriched in both HP1a and H3K9me3

Supplementary Material 8: Additional files 8 – Summary of changes in transcriptomic profile in senescence induced by doxorubicin or curcumin.

## Data Availability

Data is provided within the manuscript and supplementary information files.

## Abbreviations

ANLN: Anillin
AP: Atherosclerotic plaque
ChIP-seq: Chromatin immunoprecipitation followed by sequencing
CLDN1: Claudin-1
CTRL: Young control cells
CUR: Cells treated with curcumin
DEGs: Differentially expressed genes
DOX: Cells treated with doxorubicin
HATs: Histone acetyltransferases
HDACs: Histone deacetylases
HDMs: Histone demethylases
HMTs: Histone methyltransferases
HMGB1: High mobility group box 1 protein
HGPS: Hutchinson-Gilford progeria syndrome
HP1α: Heterochromatin Protein 1 subunitα
H3K9me3: Trimethylated lysine 9 of histone 3
H3K4me3: Trimethylated lysine 4 of histone 3
H3K27me3: Trimethylated lysine 27 of histone 3
H3K9Ac: Acetylated lysine 9 of histone 3
PCA: Principal component analysis
LMNB1: Lamin B1
PS: Premature senescence/prematurely senescent cells
PTMs: Post-translational modifications
RS: Replicative senescence/replicatively senescent cells
SIPS: Stress-induced premature senescence
TSS: Transcription start site
VSMCs: Vascular smooth muscle cells
