# Supplementary material for: Senescence-associated alterations in histone H3 modifications, HP1 alpha levels and distribution, and in the transcriptome of vascular smooth muscle cells in different types of senescence
Source: Cell Commun Signal. 2025 Jul 1;23:321. doi: 10.1186/s12964-025-02315-8 (PMC12220758; doi:10.1186/s12964-025-02315-8)
Supplement: Supplementary file 1 — Supplementary Material 1: Additional file 1 - Verification of senescence model in VSMCs in vitro and evaluation of senescence/proliferation state of VSMCs isolated from atherosclerotic plaque (ex vivo) [file 12964_2025_2315_MOESM1_ESM.docx]

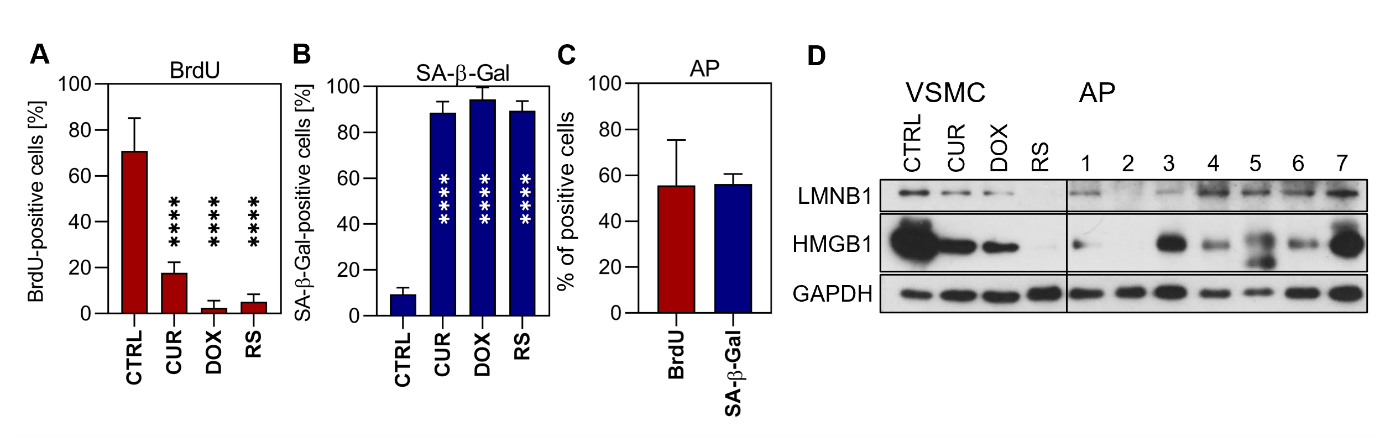


**Additional files 1.** Verification of senescence model in VSMCs *in vitro* and evaluation of senescence/proliferation state of VSMCs isolated from atherosclerotic plaque (*ex vivo*) **(A)** Percentage of dividing (BrdU-positive) VSMCs *in vitro* (n = 5). **(B)** Percentage of senescent (SA-β-gal-positive) cells in different types of VSMC senescence *in vitro* (n = 5). **(C)** Percentage of proliferating (BrdU-positive, n=5) and senescent (SA-β-gal- positive, n = 7) cells in the population of cells obtained from atherosclerotic plaque. **(D)** The representative image of the LMNB1 and HMGB1 protein expression profile analyzed by Western blotting in VSMCs *in vitro* (left panel, n = 3) and *ex vivo* (right panel, n = 7). Statistical analysis was performed using one-way ANOVA compared to control cells: (***) p < 0.001, (****) p < 0.0001

HMGB1 is a non-histone protein present in most cells, and its functions include regulating gene expression. By attaching to DNA strands, it alters the spatial structure of promoter sites to facilitate access by the transcriptional machinery [12, 13]. It can interact with multiple transcription factors and enhance their activity [14]. Previous studies have shown that expression of this protein decreased in senescence [15]. VSMCs showed a decrease in HMGB1 levels in both types of senescence, but the decrease in RS was more pronounced. The above analysis showed some similarity between PS and RS, and served mainly as a control for senescence induction. The models of VSMC senescence have been established and described by us previously [6-8].
