# Supplementary material for: Senescence-associated alterations in histone H3 modifications, HP1 alpha levels and distribution, and in the transcriptome of vascular smooth muscle cells in different types of senescence
Source: Cell Commun Signal. 2025 Jul 1;23:321. doi: 10.1186/s12964-025-02315-8 (PMC12220758; doi:10.1186/s12964-025-02315-8)
Supplement: Supplementary file 2 — Supplementary Material 2: Additional file 2 - Control selection and verification of PS and RS efficacy in fibroblasts [file 12964_2025_2315_MOESM2_ESM.docx]

**
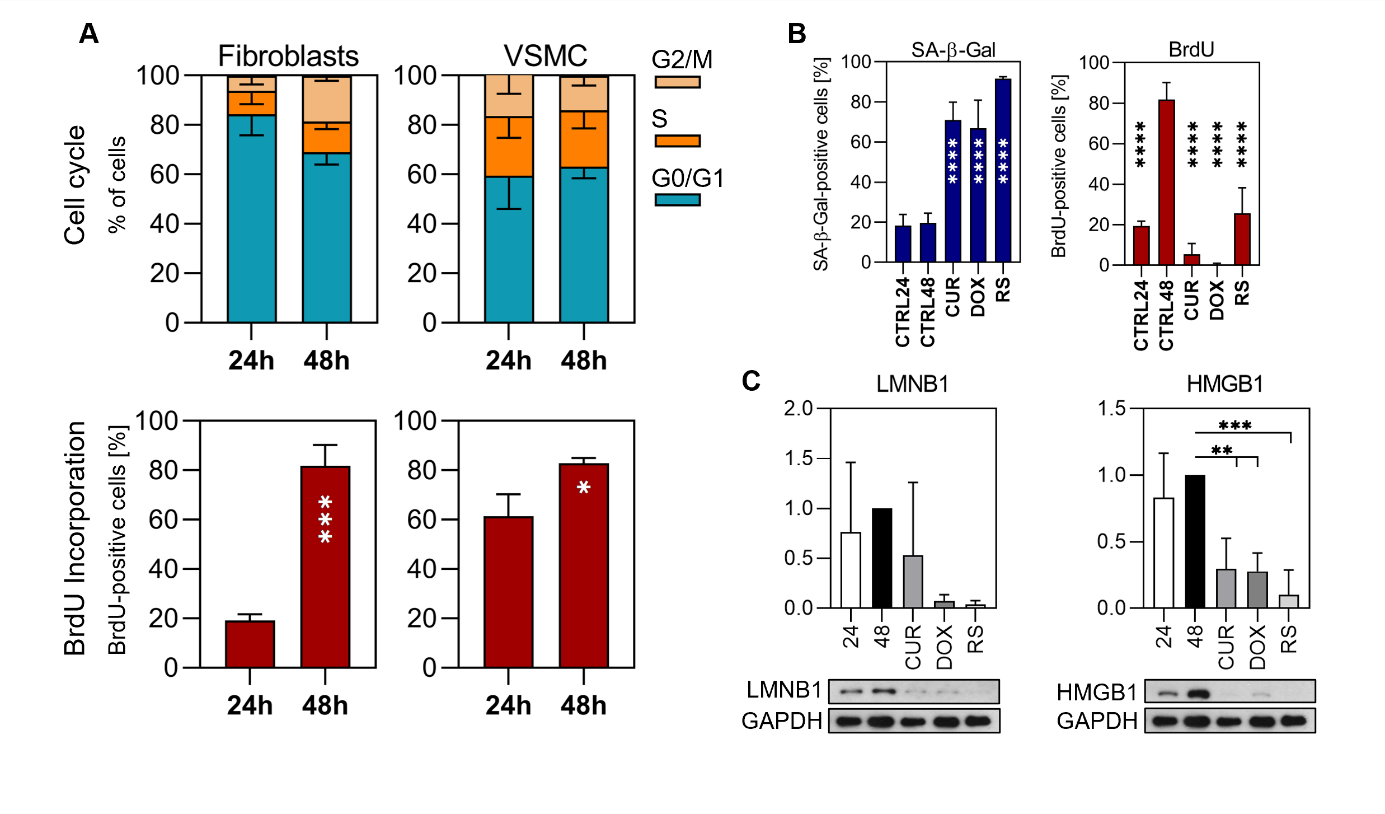
**

**Additional files 2** Control selection and verification of PS and RS efficacy in fibroblasts. **(A)** Analysis of the cell cycle of control fibroblasts (and VSMCs) harvested 24 and 48h after seeding. Evaluation of the percentage of cells in each phase of the cycle using a flow cytometer (upper panel) and comparison of the percentage of cells capable of DNA synthesis (BrdU incorporation - lower panel) in fibroblasts and VSMCs (n = 3). Statistical significance was determined using Student's t-test, (*) p < 0.05, (***) p < 0.001. **(B)** Verification of senescence model - percentage of senescent (SA-β-gal positive, n=4) and of dividing (BrdU-positive, n=4) cells in different types of fibroblasts senescence. (**C**) Densitometric analysis and representative image of HMGB1 and LMNB1 proteins level in fibroblasts analyzed by Western blotting from 3 different experiments. CTRL - young cells (24, 48 hours after seeding), DOX - doxorubicin-induced senescence, CUR - curcumin-induced senescence, RS - replicative senescence

To test whether the changes observed in VSMCs were universal or cell type-specific, selected analyses were performed on fibroblasts, a classic model for studying senescence. The use of cells 48 hours after seeding, in addition to those collected after 24 hours, was due to the observation concerning differences in the kinetics of division between VSMCs and fibroblasts, which, as we have shown, can have important implications for the final interpretation of the results. While no differences could be seen in the cell cycle phase frequency and percentage of cells capable of DNA replication 24 or 48 h after seeding in the case of VSMCs (G0/G1 – 24h 59%, 48h 63%, S – 24h 24%, 48h 23%, G2/M – 24h 17%, 48h 14%), fibroblasts matched the proliferative potential of VSMCs only after 48. Cytometric analysis revealed that fibroblasts after 24 hours post-seeding were mainly in the G0/G1 phase of the cell cycle (G0/G1 84%, S 10%, G/M 6%), while 48-hour culture displayed a higher percentage of cells entering the S and G2/M phases was detected (G0/G1 69%, S 12%, G/M 19%). Moreover, after 24-hours there appeared to be no significant differences in HMBG1 and LMNB1 protein levels between control and senescent cells. In contrast, a comparative analysis with the 48-hour control cells, showed noticeable differences.
