# Supplementary material for: Senescence-associated alterations in histone H3 modifications, HP1 alpha levels and distribution, and in the transcriptome of vascular smooth muscle cells in different types of senescence
Source: Cell Commun Signal. 2025 Jul 1;23:321. doi: 10.1186/s12964-025-02315-8 (PMC12220758; doi:10.1186/s12964-025-02315-8)
Supplement: Supplementary file 3 — Supplementary Material 3: Additional file 3 - The visual enrichment analysis in IGV software of H3K9me3 in the pericentromeric and subtelomeric regions of chromosome 7 [file 12964_2025_2315_MOESM3_ESM.docx]

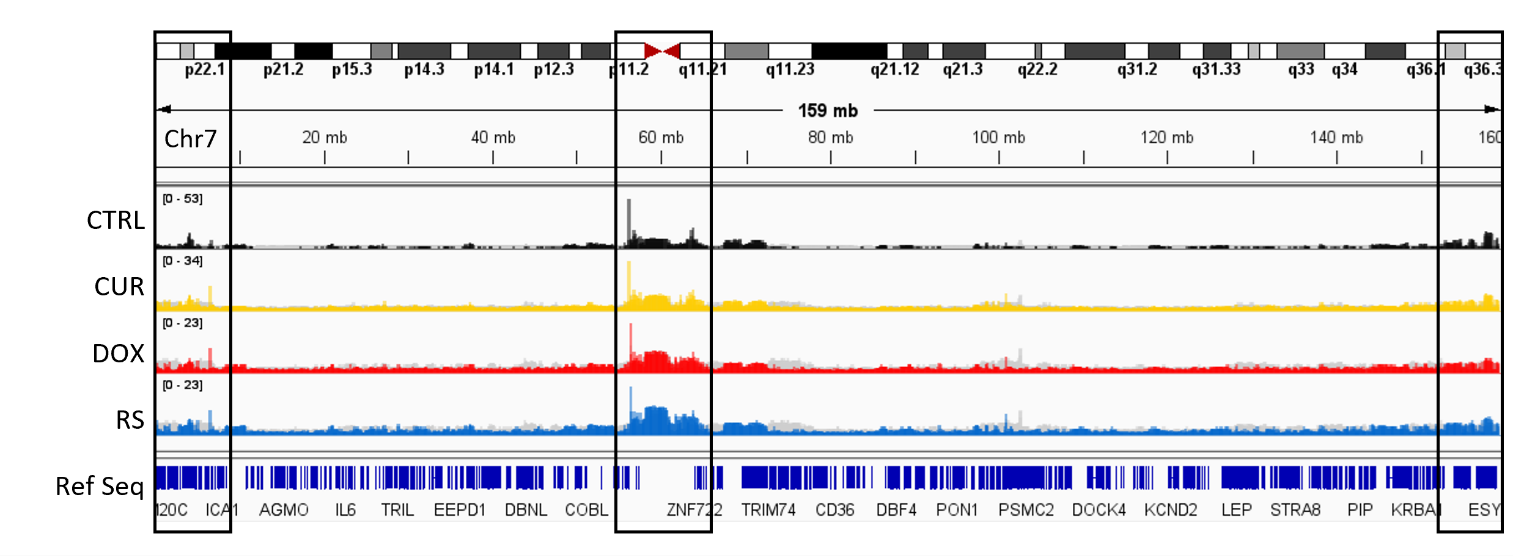


**Additional files 3** The visual enrichment analysis in IGV software. The analysis, based on merged peaks from three biological replicates, revealed a slightly higher density of H3K9me3 in the pericentromeric and subtelomeric regions of chromosome 7 (highlighted by black brackets). Experimental variants are color-coded as follows: CTRL – black, CUR – yellow, DOX – red, RS – blue input - gray.
