# Supplementary material for: Senescence-associated alterations in histone H3 modifications, HP1 alpha levels and distribution, and in the transcriptome of vascular smooth muscle cells in different types of senescence
Source: Cell Commun Signal. 2025 Jul 1;23:321. doi: 10.1186/s12964-025-02315-8 (PMC12220758; doi:10.1186/s12964-025-02315-8)
Supplement: Supplementary file 4 — Supplementary Material 4: Additional files 4 - Enrichment analysis of H3K4me3 in the promoter regions of E2F family protein-encoding genes [file 12964_2025_2315_MOESM4_ESM.docx]

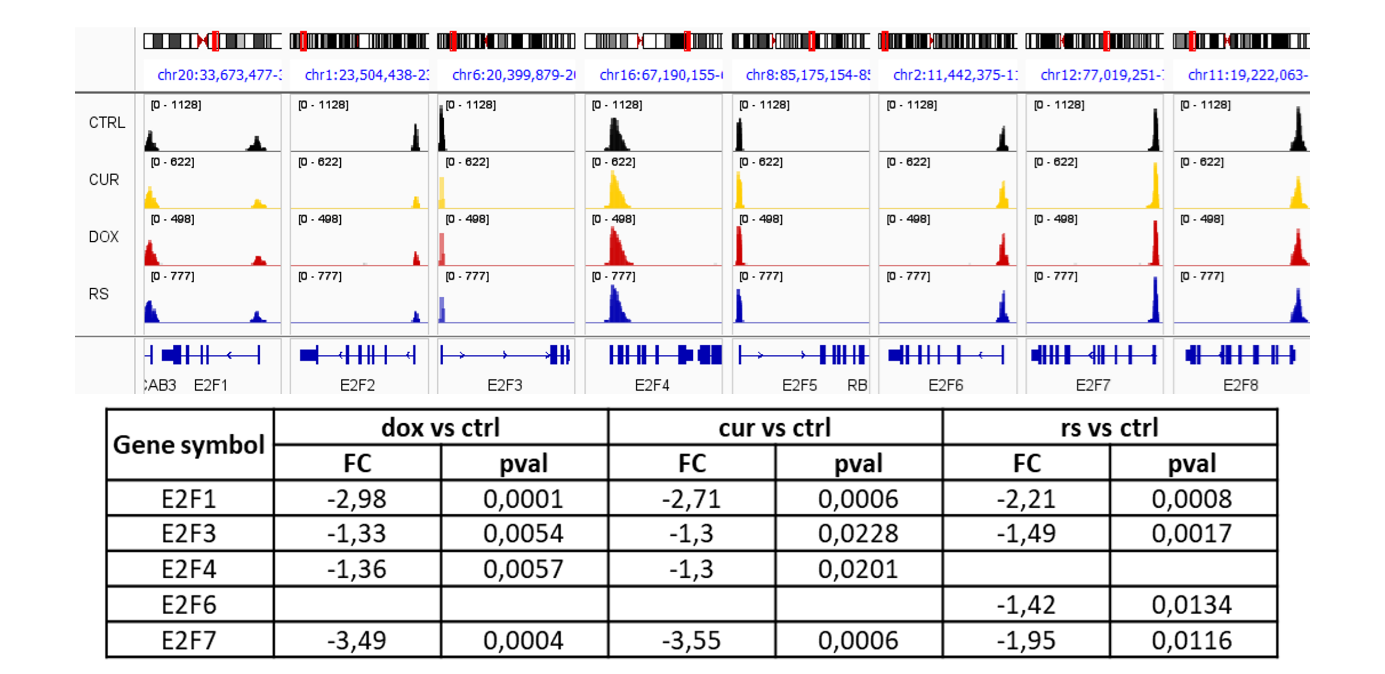


**Additional files 4.** Enrichment analysis of H3K4me3 in the promoter regions of E2F family protein-encoding genes. The upper panel shows ChIP-seq data of merged peaks from three biological replicates, with experimental variants color-coded as follows: CTRL – black, CUR – yellow, DOX – red, RS – blue, input – gray. The table below presents gene expression analysis of the E2F protein family, performed using microarrays, with results shown as fold change (FC) relative to control cells. Data analysis was conducted using TAC software
