# Supplementary material for: Senescence-associated alterations in histone H3 modifications, HP1 alpha levels and distribution, and in the transcriptome of vascular smooth muscle cells in different types of senescence
Source: Cell Commun Signal. 2025 Jul 1;23:321. doi: 10.1186/s12964-025-02315-8 (PMC12220758; doi:10.1186/s12964-025-02315-8)
Supplement: Supplementary file 8 — Supplementary Material 8: Additional files 8 – Summary of changes in transcriptomic profile in senescence induced by doxorubicin or curcumin. [file 12964_2025_2315_MOESM8_ESM.docx]

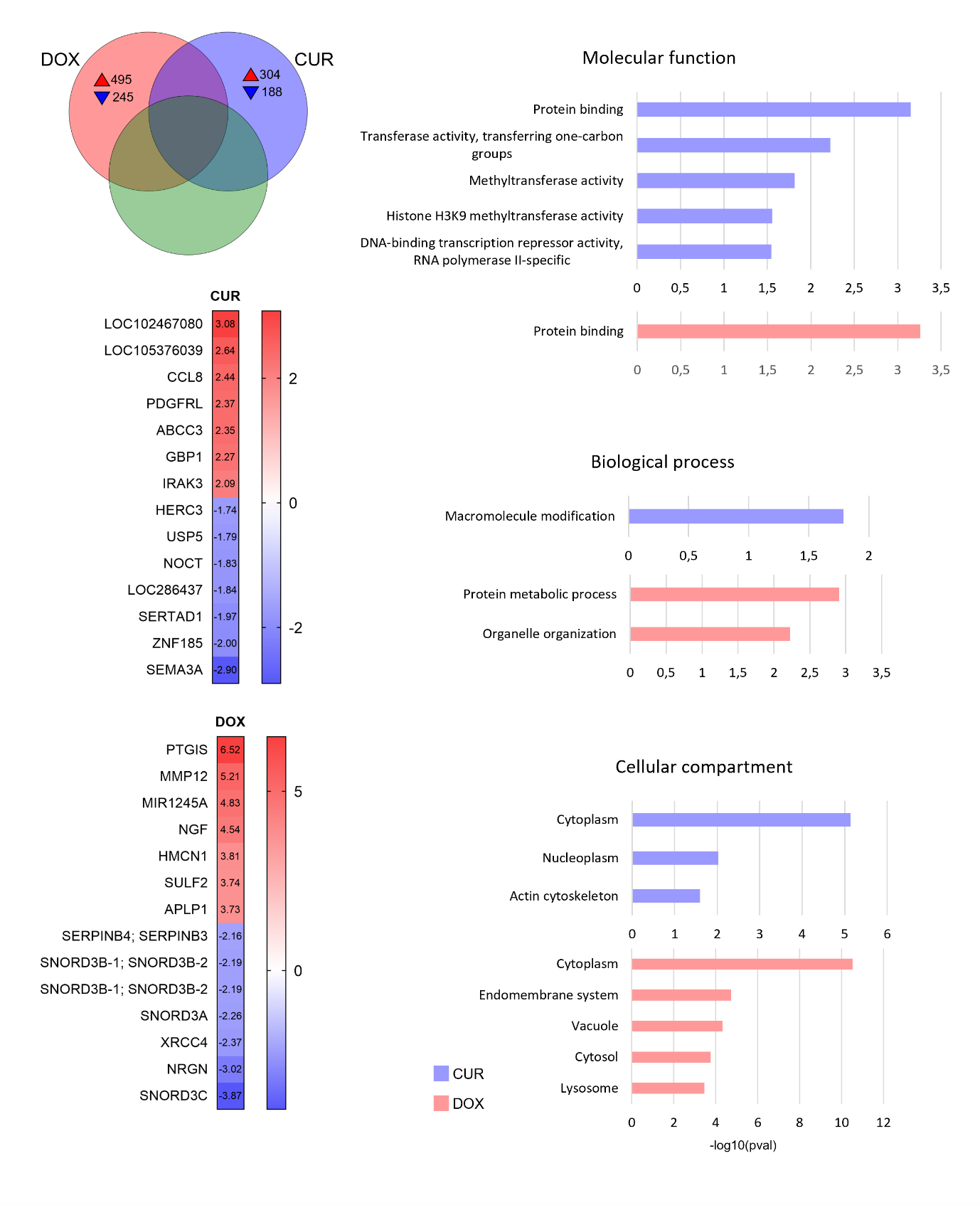


**Additional file 8.** Summary of changes in the transcriptomic profile in senescence induced by doxorubicin or curcumin. The Venn diagram shows the intersections of DEGs in each experimental variant relative to young cells, including the number of genes in the dox and cur subsets. For each type of senescent cells, the number of genes with increased (red triangle) and decreased (blue triangle) expression is indicated. The heatmap below shows the fold changes of 7 upregulated and downregulated genes characteristic of curcumin- and doxorubicin-induced senescence. The graphs on the right show a functional analysis of DEGs characteristic of DOX or CUR, including molecular function, biological processes and cellular compartments. The analysis was performed using the g:Profiler tool, which relied on the Gene Ontology database.
